# Extended correlation functions for spatial analysis of multiplex imaging data

**DOI:** 10.1017/S2633903X24000011

**Published:** 2024-02-15

**Authors:** Joshua A. Bull, Eoghan J. Mulholland, Simon J. Leedham, Helen M. Byrne

**Affiliations:** 1 Wolfson Centre for Mathematical Biology, Mathematical Institute, University of Oxford, Oxford OX2 6GG, UK; 2 Centre for Human Genetics, Nuffield Department of Medicine, University of Oxford, Oxford OX3 7BN, UK; 3 Translational Gastroenterology Unit, John Radcliffe Hospital, University of Oxford, Oxford OX3 9DU, UK; 4 Oxford NIHR Biomedical Research Centre, John Radcliffe Hospital, University of Oxford, Oxford OX3 9DU, UK; 5 Ludwig Institute for Cancer Research, Nuffield Department of Medicine, University of Oxford, Oxford OX3 7DQ, UK

**Keywords:** Digital pathology, image analysis, multiplex imaging, pair correlation function, spatial statistics

## Abstract

Imaging platforms for generating highly multiplexed histological images are being continually developed and improved. Significant improvements have also been made in the accuracy of methods for automated cell segmentation and classification. However, less attention has focused on the quantification and analysis of the resulting point clouds, which describe the spatial coordinates of individual cells. We focus here on a particular spatial statistical method, the cross-pair correlation function (cross-PCF), which can identify positive and negative spatial correlation between cells across a range of length scales. However, limitations of the cross-PCF hinder its widespread application to multiplexed histology. For example, it can only consider relations between pairs of cells, and cells must be classified using discrete categorical labels (rather than labeling continuous labels such as stain intensity). In this paper, we present three extensions to the cross-PCF which address these limitations and permit more detailed analysis of multiplex images: topographical correlation maps can visualize local clustering and exclusion between cells; neighbourhood correlation functions can identify colocalization of two or more cell types; and weighted-PCFs describe spatial correlation between points with continuous (rather than discrete) labels. We apply the extended PCFs to synthetic and biological datasets in order to demonstrate the insight that they can generate.

## Impact Statement

This paper introduces three methods for performing spatial analysis on multiplex digital pathology images. We apply the methods to synthetic datasets and regions of interest from a murine colorectal carcinoma, in order to illustrate their relative strengths and weaknesses. We note that these methods have wider application to marked point pattern data from other sources.

## 1. Introduction

The move to digital pathology is revolutionizing the way in which histological samples are processed, viewed and analyzed. Until recently, pathology was restricted to expert manual assessment of hematoxylin and eosin and immunohistochemistry (IHC) slides stained with a small number of colored dyes. Multiplex modalities now enable digital visualization of whole slide images (WSIs), stained with relatively large numbers of markers, at submicrometer resolution. Digital pathology slides can be generated using a variety of methods, including multiplex IHC, imaging mass cytometry (IMC), co-detection by indexing (CODEX/Phenocycler), and multiplexed ion beam imaging.^(^^1–4^^)^ These platforms can generate images with 50 or more cellular markers (see, e.g., Reference (5)). As the number of cell types discernible in a multiplex image increases, simply viewing an image can be challenging because of the difficulty in choosing a unique coloring for each cell marker. Additionally, existing statistical methods struggle to exploit the full range of spatial information contained within the data, with analysis dominated by nonspatial metrics such as cell counts or basic spatial metrics such as mean intercellular distances, which do not account for the wider spatial context within an image. While the methodology underlying different imaging technologies may vary, the images they generate all encode high-resolution information about the spatial location of multiple cell markers. As such, computational methods developed to analyze cell locations generated from one multiplex modality can be applied straightforwardly to data generated from another.

State-of-the-art pipelines for the statistical analysis of multiplex images typically involve at least two preprocessing steps: *cell segmentation*, in which the boundaries of individual cells are identified, and *cell classification*, in which cells are assigned to categories based on the panel of markers used for image generation.^(^^6–9^^)^ The accuracy of cell segmentation has improved significantly in recent years, driven primarily by advances in artificial intelligence (AI)-based approaches for cell detection.^(^^10^^,^^11^^)^ Many of these methods can be accessed via open source digital pathology platforms such as Qupath^(^^12^ ^)^ or MCMICRO^(^^13^ ^)^, commercial tools such as HALO (indicalab.com/halo) and Visiopharm (visiopharm.com), and standalone software such as Deepcell^(^^10^^)^ and Cellpose.^(^^11^ ^)^ By contrast, there are fewer tools for cell classification, due perhaps to variation in the panels used for a given study. Existing tools are typically iterative and semi-supervised.^(^^6^ ^,^^7^ ^,^^14^ ^)^

The above improvements in preprocessing digital pathology slides are increasing the demand for methods that can describe and quantify the spatial information contained within multiplex images. Such information is important because there is increasing evidence that physical contact can alter cell behaviors and drive disease progression. For example, the formation of tumor microenvironment of metastasis (TMEM) doorways is implicated in the metastasis of cancer stem cells.^(^^15^ ^,^^16^ ^)^ TMEMs form when a Mena^Hi^ tumor cell, a macrophage, and an endothelial cell come into physical contact on the surface of a blood vessel.^(^^17^ ^)^ This three-way spatial interaction enables tumor cells first to intravasate and then to metastasize to other parts of the body, and has also been implicated in cancer cell acquisition of a stem-like phenotype.^(^^17^ ^)^ Other biological effects that manifest in altered spatial interactions include clustering of immune cells and alveolar progenitor cells in the lungs during COVID-19 progression,^(^^7^ ^)^ and the formation of distinct cellular neighbourhoods which drive antitumoral immune responses in the invasive front of colorectal cancer. For example, neighbourhoods which are rich in both granuloctyes and PD-1 + CD4+ T cells correlate positively with patient survival.^(^^18^ ^)^ While spatially averaged statistics, such as cell counts, can be readily calculated from segmented and classified images, describing and quantifying the spatial organization of cell types requires more complex analytical tools.

One promising approach for exploiting the spatial structure of multiplex images is AI and machine learning, which learns to identify those regions of an image that are most strongly associated with clinical features such as patient prognosis and disease status.^(^^19^ ^,^^20^ ^)^ Machine learning approaches include convolutional neural networks, generative adversarial networks, and transformers. They have been used to perform a range of tasks, such as automatic identification of informative regions in WSIs,^(^^21^ ^)^ segmentation of ductal carcinoma in situ,^(^^22^ ^)^ and prediction of molecular signatures from tissue morphology.^(^^23^ ^)^ However, such machine learning methods typically require large training datasets and it can be difficult to understand or interpret their predictions. Further, machine learning methods usually require the same marker combinations to be used in each image, with data ideally collected from the same equipment; otherwise they may require retraining on additional datasets. “Interpretable” machine learning models or “explainable AI” provide potential solutions to this, but have yet to achieve widespread application.^(^^19^ ^,^^24^ ^,^^25^ ^)^

Segmented and classified multiplex images can be viewed as marked point processes, in which 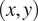 coordinates representing cell centers are labeled with a “mark” describing their cell type. Statistical and mathematical methods for analyzing these data are typically more amenable to interpretation than machine learning approaches, since they quantify interactions between specific cell populations. For example, statistics such as the mean minimum distance between two cell types provide an accessible entry point for analysis of spatial data (e.g., References (26, 27)), and are available in several software tools.^(^^12^ ^,^^28^ ^)^ Statistical approaches based on correlation metrics that were originally developed for ecological applications can also be used to determine whether pairs of cells are colocated more (or less) frequently than would be expected through random chance.^(^^7^ ^,^^29^ ^)^ By viewing a multiplex image as a network in which two cell centers are connected if the cells are in physical contact, methods from network science can be used to identify common, recurring motifs within the cell interactions.^(^^7^ ^)^ Notably, many network-based approaches use graph neural networks to analyze the spatial patterns formed by the different cell populations (see, e.g., References (30, 31)). Recently, topological data analysis (TDA), a mathematical field which quantifies the shape of datasets, has emerged as a powerful tool for characterizing histology data across multiple scales of resolution in terms of topological features such as connected components and “loops.”^(^^32^ ^,^^33^ ^)^

A range of spatial statistics can be used to analyze point processes. These include the Morisita–Horn index, which quantifies dissimilarity between two populations^(^^27^ ^,^^34^ ^)^; Ripley’s K function, which describes clustering or exclusion between points^(^^35^ ^,^^36^ ^)^; and the J-function, which identifies clustering or exclusion by computing nearest-neighbor distributions.^(^^37^ ^,^^38^ ^)^ For points with more complex, continuous marks, such as cell size or marker intensity, methods such as mark correlation functions^(^^39–42^ ^)^ or mark variograms^(^^43^ ^,^^44^ ^)^ can be used. For a detailed description of spatial statistical methods for analyzing spatial point patterns, we refer the interested reader to textbooks such as References (45–47).

While the above methods have been successfully applied to histology data, the complexity of multiplex imaging data means that there is scope for more detailed statistical and mathematical analyses which surpass what is possible with existing methods. In this paper, we focus on one spatial statistic – the cross-pair correlation function (cross-PCF) – which we use as a foundation to show how existing tools can be adapted to create new statistics that provide more detailed descriptions of multiplex imaging data. The PCF quantifies colocalization and exclusion between pairs of points, across multiple length scales. It is closely related to the cross-PCF, which identifies correlation between cells of different types. PCF approaches are useful, but their limitations restrict their wider applicability to multiplex data:

1. Cross-PCFs cannot easily resolve heterogeneity in spatial clustering within a region of interest (ROI). Variants of the cross-PCF that account for such heterogeneity do not quantify the contributions of different subregions of an ROI to its overall signal.^(^^35^ ^)^
2. Cross-PCFs can identify correlations between pairs of cells in a spatial neighbourhood, but not between three or more cell types.
3. Cross-PCFs require cell marks to be discrete, or categorical. Several alternative methods can accommodate continuous marks (e.g., References (39, 43, 44)), but are unsuitable for establishing how the spatial association between cells changes as their continuous marks vary.

In this paper, we discuss three extensions of the cross-PCF that address these limitations. The topographical correlation map (TCM) identifies heterogeneity in the correlation between pairs of cells across an ROI, and has previously been applied by us to IMC data.^(^^7^ ^)^ The neighbourhood correlation function (NCF) extends the cross-PCF to quantify the correlation between three or more different cell types. Finally, the weighted-PCF (wPCF) quantifies correlation between two cell populations where one, or both, have a continuous mark, and has been applied to synthetic data.^(^^48^ ^)^ In this paper, we present the first applications of the NCF and the wPCF to multiplex imaging data.

Other authors have attempted to address some of these limitations using methods that differ from those we propose. For example, Lavancier et al.^(^^49^ ^)^ show how to generate a map of colocalization scores based on the correlation of objects in two binary images, which could be applied to multiplex data before segmentation (in contrast to the TCM, which is developed for point data). Anselin^(^^50^ ^)^ introduced the local indicator of spatial association (LISA), which decomposes global spatial statistics into local metrics that can then be mapped onto the tissue. The TCM follows this approach, with the addition of a linearization step that enables local contributions to the cross-PCF to be combined by summing kernels at each point to form a smooth surface. Previous attempts to compute the correlation between more than two points simultaneously have also been proposed, such as the triangle-counting function^(^^45^ ^,^^46^ ^,^^51^ ^)^ and the 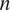 -point correlation function.^(^^52^ ^,^^53^ ^)^ In particular, the NCF adopts a similar approach to the triangle-counting function, but with the distance between three (or more) points being described by the radius of the smallest circle enclosing those points rather than the maximum distance between some pair of them (since this metric generalizes more readily to 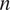 points and is sensitive to the location of all of them, rather than the most distant pair). Finally, the wPCF uses a kernel approach to permit the local contribution to the cross-PCF from each point to vary according to how closely their continuous mark matches a specified target value. This varies substantially from previous approaches to define PCF-like functions on points with continuous marks, which are typically not expressed as functions of a target mark.^(^^39^ ^,^^41^ ^,^^43^ ^,^^44^ ^)^

The remainder of the paper is structured as follows. In the methods section, we define the TCM, NCF and wPCF, and present motivating examples generated from synthetic data. We also introduce a biological dataset that derives from multiplex IHC images of a murine model of colorectal cancer.^(^^54^ ^)^ In the results section, we apply the TCM, NCF, and wPCF to this ROI, and demonstrate how each statistic identifies different properties of the spatial interactions that exist between different immune cell populations and cancer cells. We conclude by discussing how these methods expand the scope of the cross-PCF for analyzing multiplex images, and suggest possible directions for further investigation.

## 2. Methods

In this section, we introduce the synthetic and experimental datasets which we analyze in this paper. We then define the PCF and cross-PCF and their extensions: the TCM, NCF, and wPCF. The definitions are accompanied by illustrative examples based on the synthetic datasets.

### 2.1. Data

We constructed two synthetic datasets, which are used in the Methods section to develop intuition and understanding of the different spatial statistics. We also introduce a murine colorectal cancer imaging dataset, which is used in the Results section to illustrate the performance of the methods on multiplex imaging data.

#### 2.1.1. Synthetic data

##### 2.1.1.1. Synthetic dataset I

We consider two cell types, with categorical marks 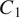 and 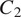 . We generate point clouds using different point processes on the left- and right-hand sides of a 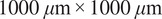 square domain (see Figures 2a and 4a). On the left half of the domain (i.e., for 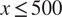 ), a Thomas point process is used to generate clustered data.^(^^55^ ^)^ This modified Neyman–Scott process samples cluster centers from a Poisson process and samples a fixed number of points from Gaussian distributions around each cluster center.^(^^56^ ^)^ In synthetic dataset I, we randomly position 20 cluster centers in 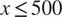 , and sample 10 points of each cell type from a 2D Gaussian distribution, with standard deviation 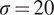 and mean 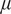 located at the cluster center. In 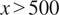 , the same process is used, but 10 cluster centers are chosen independently for each cell type, leading to a composite point pattern containing 300 cells of each type. By construction, synthetic dataset I exhibits strong colocalization between cells of types 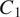 and 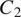 in 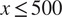 , while each cell type is located in independent clusters in 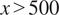 . We assign a second, continuous mark 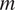 to cells of type 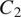 . Those with 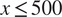 are randomly assigned a continuous mark 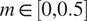 while those with 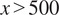 are assigned a mark 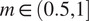 . Consequently, when a cluster contains both cell types, cells of type 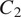 have low marks ( 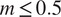 ), and when it contains only cells of type 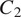 high marks ( 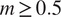 ) are present.

##### 2.1.1.2. Synthetic dataset II

The second synthetic dataset comprises two distinct point patterns, each containing cells of types, 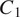 , 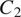 , and 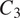 (see Figure 3). In both patterns, three cluster centers are positioned at 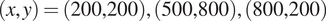 . For the first point cloud, each cluster contains 25 cells from two different cell types, with locations chosen from a 2D normal distribution (mean 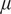 at the cluster center, standard deviation 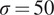 ), so that all three pairwise combinations of cell types are represented (for a total of 50 cells of each type). The same process is used to generate the second point cloud, except all three cell types are present in each cluster (i.e., a total of 75 cells of each type). By contrast, in the first pattern, no cluster contains all three cell types but each pairwise combination of cell types is present in one cluster.

#### 2.1.2. Multiplex IHC

##### 2.1.2.1. Animals

Intestinal tumor tissue from a villinCre^ER^Kras^G12D/+^Trp53^fl/fl^Rosa26^N1icd/+^ (KPN) mouse was used.^(^^54^ ^)^ Procedures were conducted in accordance with Home Office UK regulations and the Animals (Scientific Procedures) Act 1986. Mice were housed individually in ventilated cages, in a specific-pathogen-free facility, at the Functional Genetics Facility (Wellcome Center for Human Genetics, University of Oxford) animal unit. All mice had unrestricted access to food and water, and had not been involved in any previous procedures. The strain used in this study was maintained on C57BL/6 J background for 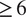 generations.

##### 2.1.2.2. Multiplex immune panel and image preprocessing

Akoya Biosciences OPAL Protocol (Marlborough, MA) was employed for multiplex immunofluorescence staining on FFPE tissue sections of 4- 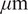 thickness. The staining was performed on the Leica BOND RXm auto-stainer (Leica Microsystems, Germany). Six consecutive staining cycles were conducted using primary antibody-Opal fluorophore pairs. The marker panel used is shown in Table 1.

**Table 1.** List of markers and opals used in the multiplex panel

Marker	Opal
Ly6G (1:300, 551459; BD Pharmingen)	Opal 540
CD4 (1:500, ab183685; Abcam)	Opal 520
CD8 (1:800, 98941; Cell Signaling)	Opal 570
CD68 (1:1200, ab125212; Abcam)	Opal 620
FoxP3 (1:400, 126553; Cell Signaling)	Opal 650
E-cadherin (1:500, 3195; Cell Signaling)	Opal 690

The tissue sections were incubated with primary antibody for an hour, and the BOND Polymer Refine Detection System (DS9800, Leica Biosystems, Buffalo Grove, IL) used to detect the antibodies. Epitope Retrieval Solution 1 or 2 was applied to retrieve the antigen for 20 min at 100 °C, in accordance with the standard Leica protocol, and, thereafter, each primary antibody was applied. The tissue sections were subsequently treated with spectral DAPI (FP1490, Akoya Biosciences) for 10 min and mounted with VECTASHIELD Vibrance Antifade Mounting Medium (H-1700-10; Vector Laboratories) slides. The Vectra Polaris (Akoya Biosciences) was used to obtain whole-slide scans and multispectral images (MSIs). Batch analysis of the MSIs from each case was performed using inForm 2.4.8 software, and the resultant batch-analyzed MSIs were combined in HALO (Indica Labs) to create a spectrally unmixed reconstructed whole-tissue image. Cell segmentation and phenotypic density analysis was conducted thereafter across the tissue using HALO.

### 2.2. ROI overview

**Figure 1.**
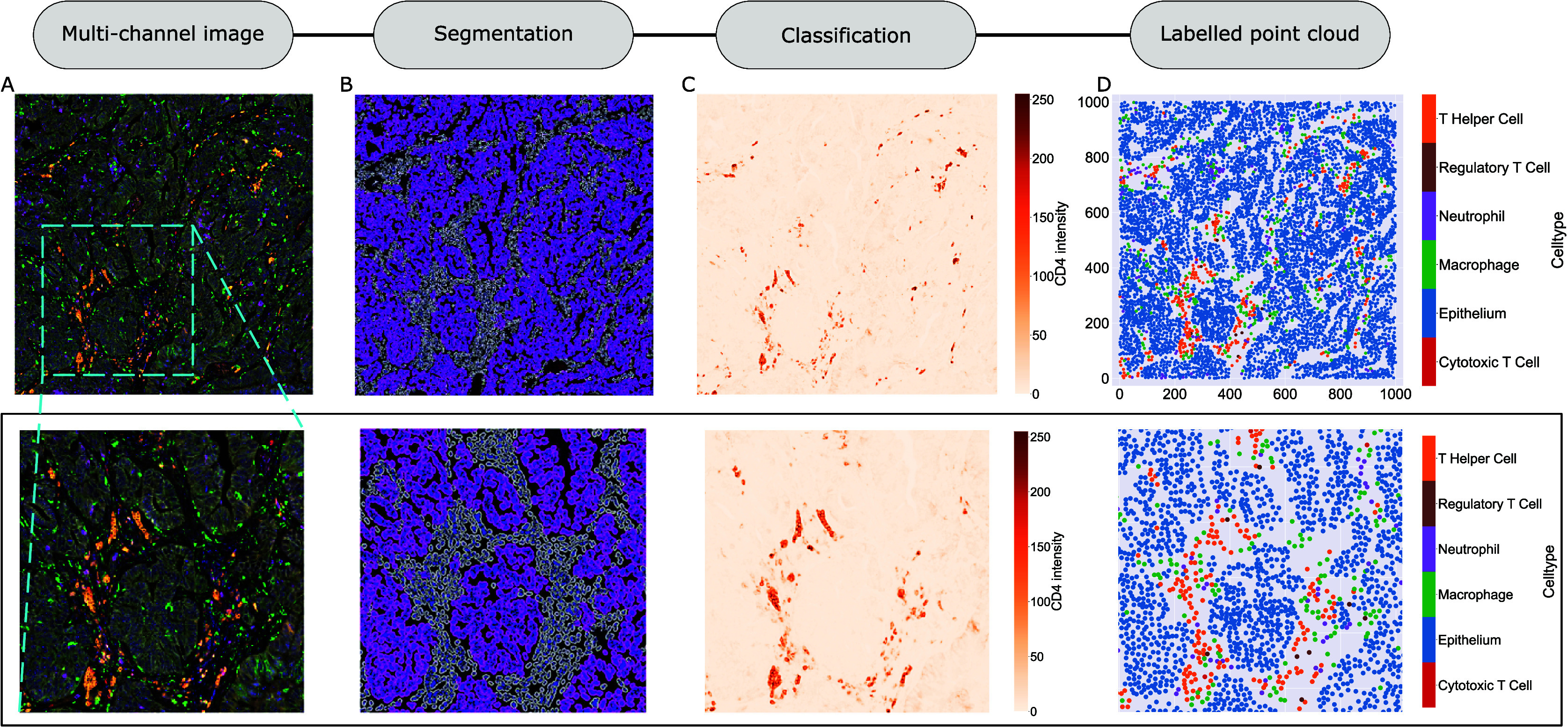
Obtaining point cloud data from a multiplex image. (a) 1 mm × 1 mm ROI from a multiplex IHC image of murine colorectal carcinoma (blue – DAPI; orange – CD4; green – CD68; magenta – Ly6G; maroon – FoxP3; red – CD8; white – E-cadherin). The epithelial cells (E-cadherin+) are cancer cells which form dense “tumor nests” that are surrounded by stromal regions. Immune cells are largely restricted to the stroma between tumor nests, so the region shows spatial correlation between immune cell subtypes (particularly macrophage, neutrophil, and T helper cell) within the stroma, and anticorrelation between immune cells and epithelial cells. (b) Cell segmentation (HALO) for the region in panel a. The edges of E-cadherin positive cells are shown in pink to aid comparison with panel a. (c) Pixel intensity from the color channel corresponding to the CD4 stain only. (d) Composite point cloud formed by classifying each cell type stained in panel a, with points placed at the centroids of segmented cells. Lower row: Magnified 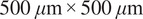 zoom from the upper panels.

We consider a 1 mm × 1 mm ROI from a KPN mouse intestinal tumor, shown in Figure 1a (three additional regions from this tumor are included in the Supplementary Material). Each color channel corresponds to a different marker (blue – DAPI; orange – CD4; green – CD68; magenta – Ly6G; maroon – FoxP3; red – CD8; white – E-cadherin). To obtain a labeled point cloud, individual cell boundaries were identified via cell segmentation (HALO, panel b). Classification of cell types was achieved by considering the average pixel intensity within a cell boundary for each marker individually (e.g., CD4 pixel intensity, panel c), with combinations of cell markers defining different cell types as outlined in Table 2. The final marked point pattern (panel d) was obtained by assigning cell labels to the centroids associated with each cell boundary.

**Table 2.** Cell types present in the ROI, with markers and number of cells present. Note that all cells must also contain sufficient DAPI staining to be classified as a cell. Due to low numbers of cytotoxic T cells and regulatory T cells, we exclude them from subsequent analyses

Cell type	Marker	Cell number
Epithelium	E-cadherin	5845
Macrophage	CD68	392
T helper cell	CD4+ FoxP3-	314
Neutrophil	Ly6G	214
Cytotoxic T cell	CD8	12
Regulatory T cell	CD4+ FoxP3+	8

The ROI in Figure 1 was selected because of the clear separation between the spatial position of immune cell subtypes and tumor nests (epithelial cells), with immune cells located predominantly in regions between epithelial cell islands. Table 2 summarizes the different cell types, the markers used to define them, and the number of cells of each type in the ROI.

### 2.3. Spatial statistics

We consider a point pattern in a rectangular domain 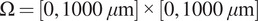 . The point pattern comprises 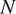 points (or cells). Cell 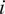 ( 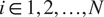 ) has spatial location 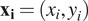 , and a set of marks which may be categorical (e.g., a label for a cell type, or a true/false label indicating whether a cell’s average stain intensity exceeds a threshold value), or continuous (e.g., the average stain intensity of a particular mark within a cell). For clarity, we denote categorical and continuous marks by 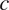 and 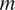 , respectively. We use lowercase for marks associated with a particular point and uppercase for target values. We introduce the indicator function 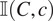 to determine whether a categorical mark associated with a point matches a target mark:
(1)

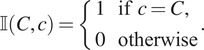

When we define correlation functions below, we will need to determine whether two points are separated by a distance “close to” 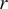 . We do this by defining an indicator function, 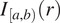 , which identifies whether the distance 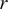 is within an interval 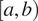 :
(2)

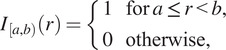

where 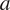 and 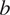 are the real numbers with 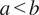 . We calculate the statistics below at a series of discrete points 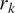 , which is equivalent to considering a sequence of annuli of width 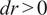 whose inner radii are separated by 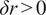 , with 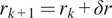 and 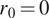 (if 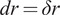 then the annuli are nonoverlapping). We denote by 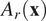 the area of the annulus with inner radius 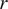 and width 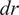 centered at the point 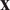 , intersected with the domain. If this annulus lies wholly inside the domain then 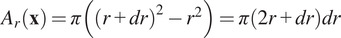 ; otherwise, only the area contained within the domain is recorded.

It is important to distinguish between the *theoretical* forms of correlation functions, which relate to properties of a point process which has generated a pattern, and the *empirical* forms of the same functions, which relate to observations of data (regardless of whether that data are generated from an underlying point process). In the definitions below, we consider only empirical versions of these functions, which may be defined differently (e.g., by using different kernels or edge-correction terms): for a detailed discussion of the differences between empirical and theoretical spatial statistics, we refer the interested reader to textbooks such as Reference (45).

It is important to note that these functions cannot distinguish, in a technical sense, between colocalization of cells due to co-intensity (points being found in the same region due to, e.g., the tissue being partitioned into tumor and stromal regions) or correlation (points being found in the same region because they are subject to the same reference process). Since cell location data are not generated by a well-defined statistical process, statistical correlation and co-intensity cannot be readily distinguished using multiplex imaging data, and we use the terms interchangeably throughout this manuscript. We note also that, in this manuscript, we use statistics to illustrate their potential as tools to guide quantitative analysis of multiplex imaging. In order to assess the significance of these (or other) spatial statistics, appropriate significance testing should be performed. For a given statistic, this could be achieved by, for example, generating a simulation envelope using data derived from CSR and comparing this with the observed measurements.

#### 2.3.1. Pair correlation function

##### 2.3.1.1. Aims

The PCF, 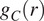 , quantifies spatial clustering or exclusion between pairs of points separated by a distance 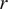 within an ROI, compared to a suitably selected null distribution. While a range of null distributions could be considered (e.g., using a Matérn hard core process to simulate randomly distributed cell centers separated by a minimum distance to approximate a cell radius^(^^57^ ^)^), we assume the null distribution is complete spatial randomness (CSR) as represented by a homogeneous spatial Poisson point process with intensity 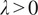 chosen to match the intensity of the point pattern being analyzed.

##### 2.3.1.2. Definition

Let 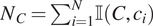 be the number of points in 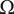 with 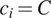 , for some categorical mark 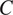 . The empirical PCF, 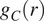 , is defined as follows:
(3)

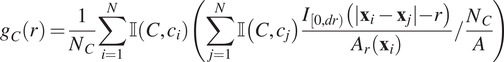

where 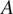 is the total area of the domain 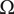 and 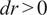 . There are many ways to account for edge effects associated with points close to the domain boundary, although the choice of a particular method is generally not critical (see, e.g., Reference (45) for a detailed discussion of this). Throughout this paper, we account for them by adjusting the contribution of each point to account for the area of each annulus contained within the domain, 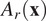 . This form of edge correction ensures that the local contribution to the PCF of a given point is based on the ratio of the observed number of points to the area of the annulus that falls within the domain; note that many other forms of edge correction are used throughout the literature,^(^^45^ ^)^ and can be substituted here without substantially changing the methods introduced below.

For a theoretical PCF, CSR generates a value of 1. We note from Equation (3) that, for the empirical PCF, 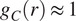 for data generated under CSR. Further, if 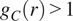 then points separated by distance 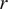 are observed more frequently than expected under CSR and we say that points at this length scale are clustered relative to CSR. Similarly, 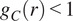 indicates fewer points than expected and is interpreted as exclusion at length scale 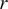 .

The structure of Equation (3) provides the basis for the generalizations of the PCF introduced below.

#### 2.3.2. Cross PCF

##### 2.3.2.1. Aims

The cross-PCF describes the correlation between pairs of points separated by distance 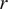 which may have different categorical labels.^(^^45^ ^)^

##### 2.3.2.2. Definition

Consider the categorical marks 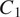 and 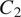 . The cross-PCF, 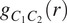 , is defined as follows:
(4)

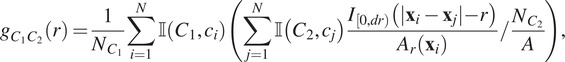

where 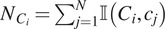 is the number of points with mark 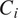 . We note that when 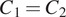 , Equation (4) reduces to Equation (3) (i.e., the cross-PCF reduces to the PCF).

##### 2.3.2.3. Example

The interpretation of the cross-PCF is similar to that for the PCF, with 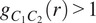 indicating correlation between points with marks 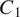 and 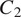 separated by distance 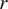 and 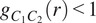 indicating exclusion at distance 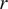 .

**Figure 2.**
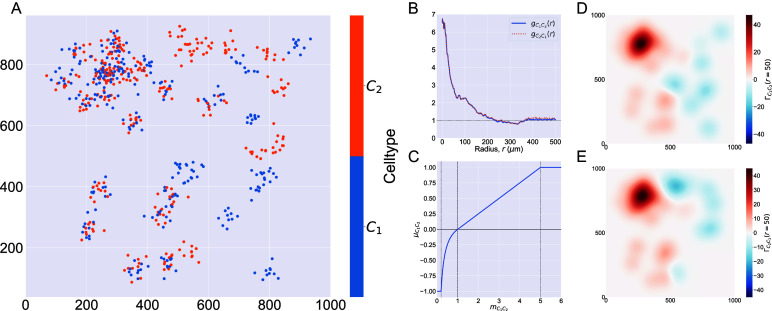
Motivating example I: Cross-PCF and topographical correlation map. (a) Synthetic dataset I: a synthetic point pattern involving two cell types, with labels 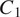 and 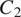 . For 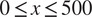 , points with labels 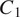 and 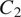 cluster together; for 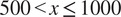 , points of types 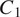 and 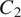 form distinct, homogeneous clusters. (b) The cross-PCFs 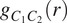 and 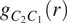 for the point pattern in panel a. The cross-PCF detects the short range clustering between cells of types 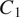 and 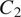 , which is present for 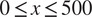 . The cross-PCFs are almost identical, differing only for large 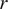 because of boundary correction terms. (c) Function used to linearize the mark 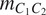 in Equation (6), used to calculate the TCM, for 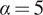 . Dashed lines represent 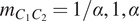 , which correspond to the maximum detectable exclusion, CSR, and the maximum detectable clustering. (d, e) TCMs 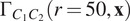 and 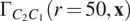 . The TCM identifies colocalization between cells of types 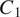 and 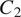 in 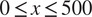 , and distinguishes between the dense cluster in the top left quadrant and smaller clusters in the bottom left quadrant. The TCM also identifies exclusion between the two cell populations in 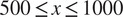 and shows this to be less pronounced than the clustering in 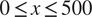 . Note that while the regions of positive correlation are similar between panels d and e, the regions of negative correlation differ.

In Figure 2, we compute two cross-PCFs for synthetic dataset I. In Figure 2a, cells with labels 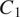 and 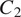 are strongly spatially correlated on the left half of the domain, while they are clustered separately on the right half. Figure 2b shows the cross-PCFs 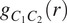 and 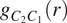 for this point pattern. Colocalization between the cell types is identified for 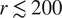 . The cross-PCFs are almost identical, since the cross-PCF is symmetric up to boundary correction terms. While the cross-PCF successfully identifies the presence of clustering between the two cell types, it does not provide information about differences in colocalization on the left- and right-hand sides of the domain.

#### 2.3.3. Topographical correlation map

##### 2.3.3.1. Aims

The TCM, 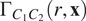 , is an example of a LISA^(^^50^ ^)^ and was introduced by us in Reference (7) to visualize spatial heterogeneity in the correlation between pairs of points across an ROI. In contrast to direct visualization of two point patterns, the TCM provides a quantitative summary of colocalization between the points which is spatially resolved across the ROI. Local maxima and minima of the TCM identify areas where points with different labels are (positively or negatively) correlated, relative to a baseline of CSR. Motivated by Equation (4), each point with mark 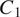 is assigned a value that quantifies its correlation with points with mark 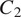 . A series of kernels centered at each point with mark 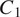 is summed to produce a spatial map of local correlations between the cell types. We note that, since these kernels are centered on points marked 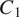 , the TCM is not symmetric (i.e., 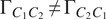 if 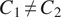 ).

##### 2.3.3.2. Definition

The TCM, 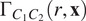 , is visualized at a specific length scale 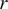 , chosen to reflect the length scale at which one wishes to observe correlation. The choice of length scale can be determined from the corresponding cross-PCF 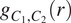 , by identifying the value at which 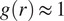 , for example, or based on *a priori* assumptions about biological behavior, for example by choosing a length scale associated with the approximate size of the cells of interest. Unless stated otherwise, we fix 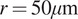 which corresponds to clustering on the length scale of two to three cell diameters. We associate a continuous mark 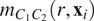 with each cell 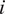 with mark 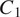 , such that
(5)

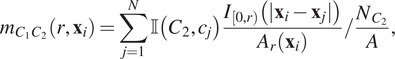

where 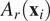 is the area of that part of the circle with radius 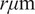 centered at 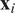 that falls within the ROI. 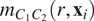 can be viewed as the contribution of each point 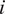 to the cross-PCF, 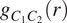 , for the special case of an annulus with inner radius 0 and width 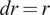 (note that this represents the cumulative contributions of the annuli used to calculate the cross-PCF up to distance 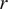 ; that is, the contribution to the K-function – see, e.g., Reference (45)). Thus, 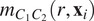 is interpreted similarly to the cross-PCF: 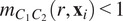 indicates anticorrelation between cells with marks 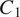 and 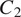 separated by a distance of at most 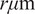 , and 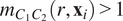 indicates correlation.

Since 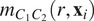 is based on a ratio of observed counts to counts expected under CSR, its interpretation is nonlinear: an observation of three times as many points as expected corresponds to 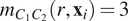 , while three times fewer points than expected leads to 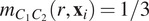 . To facilitate interpretation, we rescale 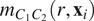 to produce a transformed mark 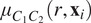 in which clustering and exclusion can be compared on a linear scale, with 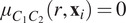 when 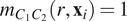 :
(6)

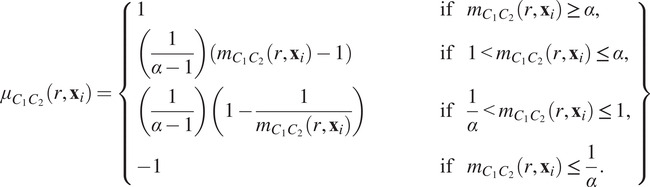

In Equation (6), the constant 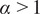 describes the maximal degree of clustering (or exclusion) which can be resolved under this transformation. A sketch of Equation (6) is presented in Figure 2c, for 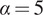 (henceforth, we fix 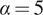 ).

After calculating 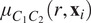 for each cell with mark 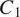 , we center a Gaussian kernel with standard deviation 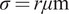 , and scaled by 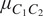 , at 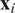 (examples showing the effect of varying 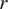 and 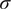 on the TCM are presented in the Supplementary Material). The TCM, 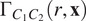 , is obtained by summing over all cells with mark 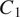 in the domain:
(7)

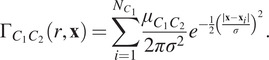

Regions in which the TCM is positive indicate that more points marked 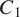 are positively correlated with points marked 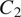 in this area than would be expected under CSR, at length scales up to 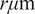 . Similarly, the TCM is negative in regions where points with mark 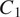 are negatively correlated with points with mark 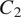 . The choice of 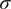 changes the resolution of the TCM; we choose 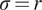 so that the resolution of the TCM approximately matches the maximum radius at which correlation contributes to the TCM (see the Supplementary Material for further details).

##### 2.3.3.3. Example

Figure 2d,e shows the TCMs associated with synthetic dataset I (the point pattern in Figure 2a) for 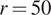 . Panel d shows 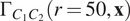 and panel e shows 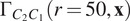 . Both TCMs identify differences in the colocalization of the two cell types on the left and right sides of the domain. In particular, 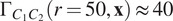 in the upper left quadrant of panels d and e, indicating strong positive correlation, with weak association in the lower left ( 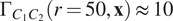 ). (Note that nonzero values of 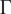 are consistent with clustering or regularity. In practice, however, significance testing should be conducted before concluding that the observed value is significantly different from 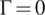 .) For 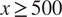 both TCMs correctly identify the regions in which cells of types 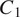 and 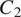 appear independently from one another ( 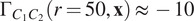 ). The cross-PCFs in panel b are dominated by the correlation on the left-hand side of the domain, and are unable to resolve the heterogeneity in clustering between the left and right sides of the domain. We note that 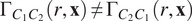 , since the kernels used to construct the TCM are centered on cells with label 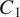 (and vice versa). While areas in which cells with mark 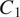 and mark 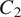 are co-located are identified by positive values of both 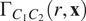 and 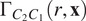 , their values differ in regions where one or other TCM is negative, as in these regions the cell densities vary (e.g., on the right-hand side of panels d and e). We, therefore, emphasize that 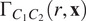 provides a spatial map of subregions in which cells with mark 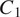 are correlated (or anticorrelated) with cells with mark 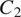 . Finally, we note that the TCM is not a density map showing the presence (or absence) of the cell types individually; for example, when 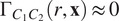 , either cells of type 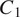 are absent, or cells of both types are present in numbers consistent with CSR.

#### 2.3.4. Neighbourhood correlation function

##### 2.3.4.1. Aims

The NCF 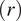 extends the PCF to quantify spatial colocation between three or more cell types with different categorical marks. We compare the observed number of triplets of points with marks 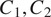 and 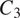 within a neighbourhood of size 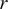 against the number of triplets expected under CSR. Selecting an appropriate definition for such a neighbourhood is nontrivial: while it is straightforward to calculate the Euclidean distance between two points, many metrics can be used to calculate the proximity of three or more points. We require a metric that is interpretable and extends naturally to more than three points. Metrics such as the area of the polygon spanning the points are unsuitable (the area of the polygon is identically zero when all points fall on a straight line, even though the points could be far apart). We consider the minimum enclosing circle (details below) as it requires all cells to lie within a “neighbourhood” of each other (with the distance between any two points at most 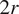 , where 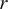 is the radius of the minimum enclosing circle). While some methods instead consider the maximum pairwise distance between any two of the points to define the distance between a set of points (see, e.g., Reference (45)), this is sensitive only to the location of the pair of points separated by the largest distance, and not to the location of other points in the set. The radius of the minimum enclosing circle can be interpreted in terms of pairwise distances (it is the length that minimizes the largest distance of any point from a common location, the center of the circle), but has a more natural interpretation in biological imaging contexts as the radius of the region in which the cells of interest are located.

##### 2.3.4.2. Definition

Consider a point pattern for which there are 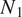 , 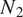 , and 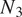 points with categorical marks 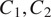 , and 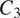 , respectively. We say that three points from this pattern fall within a “neighbourhood” of radius 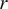 if there is a circle of radius 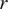 which encloses all three points. For a given set of three points 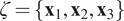 , let 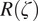 be the radius of the smallest circle enclosing every point in 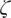 (the “minimum enclosing circle”).

There are 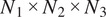 possible triplets containing one point with each mark. We calculate 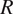 for each of these, and then determine the number of circles of radius 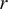 containing a unique grouping of cells with each mark (as for the PCF, these values are grouped into discrete bins of width 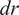 ).

As for the PCF, we compare the number of minimum enclosing circles with radius 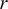 with the number expected under CSR. The probability of three points lying within a neighbourhood of radius 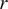 , 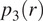 , is:
(8)

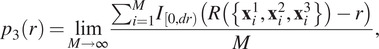

where 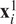 , 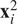 , and 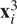 are three points within the domain, sampled under CSR. Since sampling such points is computationally cheap, 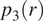 can practically be approximated for an arbitrary domain by sampling a large number of random triplets (in this section, we use 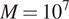 ) and calculating their minimum enclosing circles. There are many standard algorithms for computing the radii of minimum enclosing circles (see, e.g., Reference (58), or (59) for the algorithm we use).

For a point pattern containing 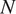 points, the NCF is defined as the ratio of the observed number of smallest neighbourhoods of radius 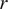 to the number of such neighbourhoods expected under CSR, 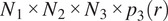 :
(9)

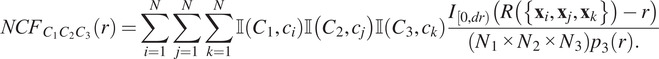



We note that it is straightforward to extend the NCF for 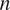 categorical marks:
(10)

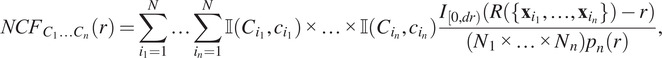

where 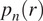 is the probability that 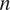 points sampled under CSR fall within a minimum enclosing circle of radius 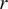 .

##### 2.3.4.3. Example

As for the PCF, 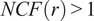 indicates clustering and 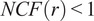 indicates exclusion. We interpret the length scale 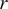 associated with the NCF as the neighbourhood radius within which the points are contained.

**Figure 3.**
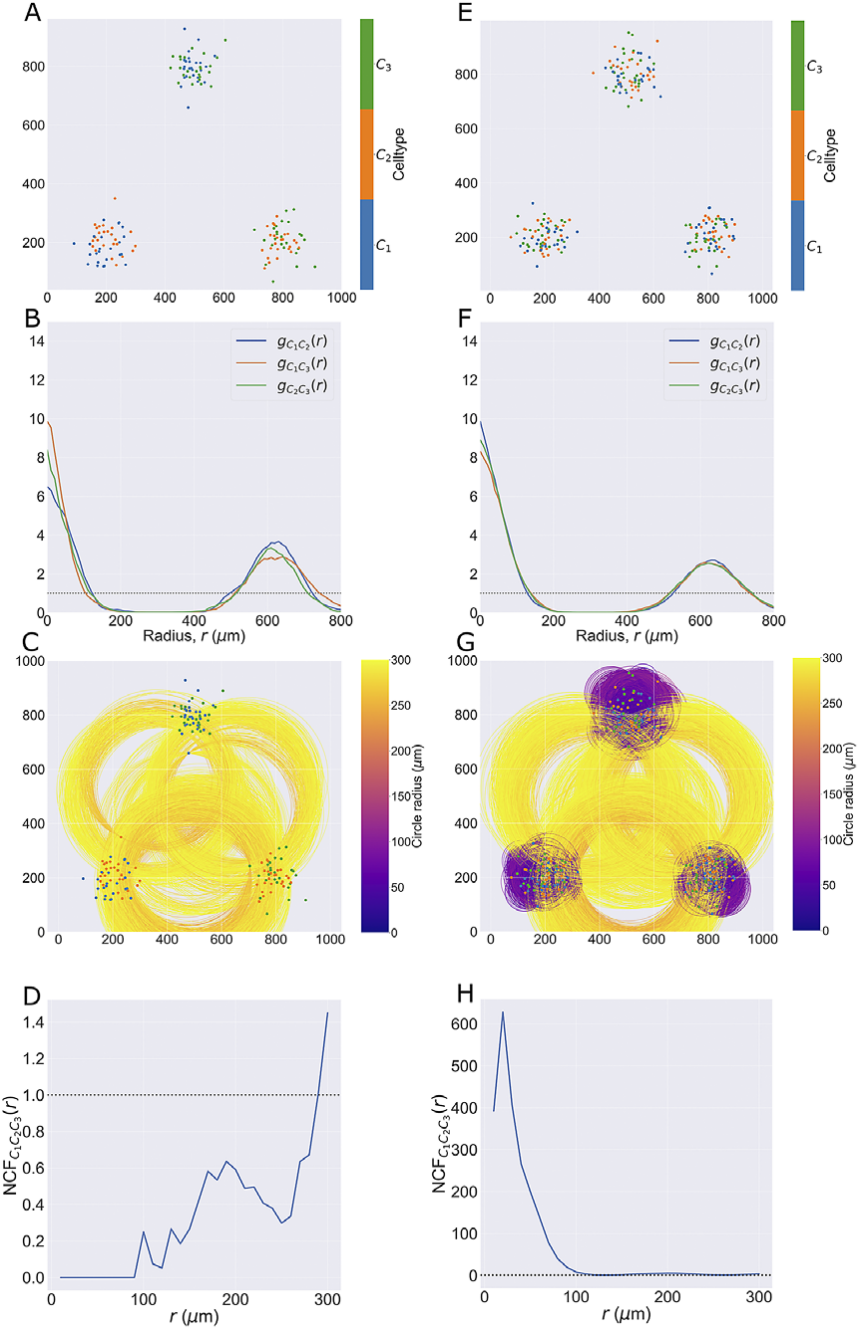
Motivating example II: Neighbourhood Correlation Function. (a, e) Synthetic dataset II: point patterns in which three cell types are spatially correlated pairwise (a) or in triplets (e). In (a), each cluster contains only two cell types, so that all three cell types are never in close proximity. In (e), all three cell types are in close proximity in each cluster. Hence, in both point patterns, there is positive correlation between pairwise combinations of cell types, but the three-way correlations differ between the panels. (b, f) Cross-PCFs for the point patterns in panels a and e, respectively. These cross-PCFs appear identical, showing strong short-range correlation between the cell types (inside a cluster), exclusion from 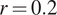 to 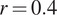 , and a second peak of correlation around 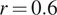 (between clusters). (c, g) Minimum enclosing circle for every combination of three points with marks 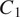 , 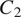 , and 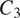 (up to circles with a radius of 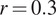 ). Circles with small radii arise when all three cell types are in close proximity (panel g). Circles are colored according to their radius. (d, h) NCFs for the point patterns in panels a and e, respectively. The NCF in panel d correctly identifies short-range exclusion between the three cell types in panel a, while the NCF in panel h identifies strong short-range correlation between the three cell types.

In Figure 3, we compare the cross-PCFs and 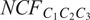 for the two point patterns from synthetic dataset II. In Figure 3a, each cluster consists of only two cell types, so that any pairwise combination of cell types can be found in close proximity while all three cell types are never in close proximity. In Figure 3e, each cluster contains all three cell types.

Figure 3b,f shows that, for synthetic dataset II, all cross-PCFs 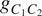 , 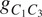 , and 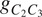 have the same shape and, hence, that pairwise correlation is insufficient to distinguish the two point patterns in this dataset. Figure 3c,g shows all minimum enclosing circles (with radius up to 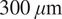 ) for these point patterns, colored according to the radius of the circle; note that when all three cell types are present within the same cluster, there are a large number of small (purple) circles present. Figure 3d shows that, in this point pattern, all three cell types are never observed within a circle of radius 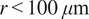 . The NCF increases from 0 to 1 as 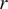 increases from 100 to 300, showing that circles with these radii may contain more triplets of cells of type 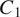 , 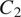 , and 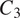 . In particular, these represent combinations of cells drawn from multiple clusters, requiring a large neighbourhood to encompass all three cell types and, hence, showing that the three do not often occur in close proximity of one another. In contrast, Figure 3h shows that the NCF distinguishes the two point patterns, by identifying strong correlation between the three cell types in neighbourhoods with radii of at most 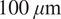 for the three-way correlation point pattern, which corresponds to the approximate radius of the clusters.

#### 2.3.5. Weighted-PCF

##### 2.3.5.1. Aims

The wPCF extends the cross-PCF to describe correlation and exclusion between cells marked with labels that may be categorical or continuous. Here, we focus on pairwise comparisons between points marked with a categorical label (e.g., points of type 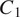 ) and those marked with a continuous label (e.g., points with mark 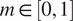 . The wPCF can also compute correlations between points labeled with two continuous marks (see Reference (48) for an example of this).

##### 2.3.5.2. Definition

Consider a set of points labeled with categorical marks ( 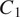 ) and continuous marks ( 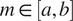 for some 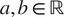 ). The wPCF describes the correlation between points with a given target mark 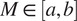 and those with a categorical mark 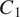 , at a range of length scales 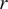 .

The cross-PCF cannot be calculated for such points since, for a continuous mark, 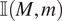 is zero almost everywhere. As such, we replace 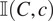 with a generalized version, the “weighting function” 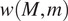 , to account for values of continuous marks 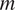 that are “close to” a target mark 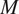 in the following way:
(11)

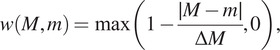

where the positive parameter 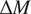 determines the width of the function’s support. Many other functional forms could be used. Following,^(^^48^ ^)^ we use a triangular kernel due to the simplicity of the relationship between 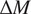 and the support of the weighting function (any marks more than 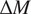 from the target mark have weight 0). We note that other kernels, such as a Gaussian kernel, could also be used, but that those that have compact support, 
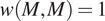
 and 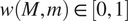 , are likely to be most informative. Choosing an appropriate value for 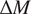 is important, and depends on the range over which the target marks vary and the desired ratio of signal to noise. In general, fixing 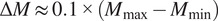 appears to provide a good balance, where 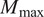 and 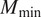 are the extremal values of the target marks (see Reference (48) for details of alternative functional forms and a detailed analysis of how the choice of weighting function and 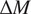 influence the signal to noise ratio in the resulting wPCF).

The wPCF is defined as follows:
(12)

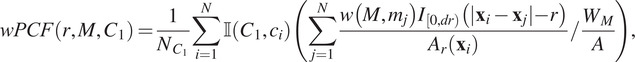

where 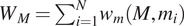 is the total “weight” associated with the target label 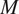 across all points. The wPCF extends the cross-PCF by weighting the contribution of each point based on how closely its continuous mark matches the target mark.

We note that the wPCF can be used to compare point clouds with two continuous marks by replacing the categorical target mark 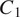 with a second continuous mark (say, 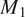 ):
(13)

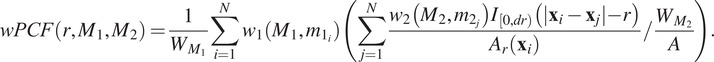



In Equation (13) the weighting functions 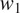 and 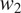 quantify proximity to target marks 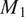 and 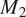 , respectively. Note that since the ranges of the marks 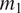 and 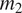 may differ substantially, the functions 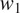 and 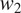 may not necessarily use the same value of 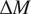 in Equation (11) (e.g., see Reference (48)).

##### 2.3.5.3. Example

We again use synthetic dataset I, where points with 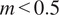 are on the left-hand side of the domain, and have been placed in clusters with the 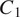 cells. In contrast, points on the right-hand side have 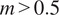 and cluster independently from the 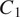 clusters.

**Figure 4.**
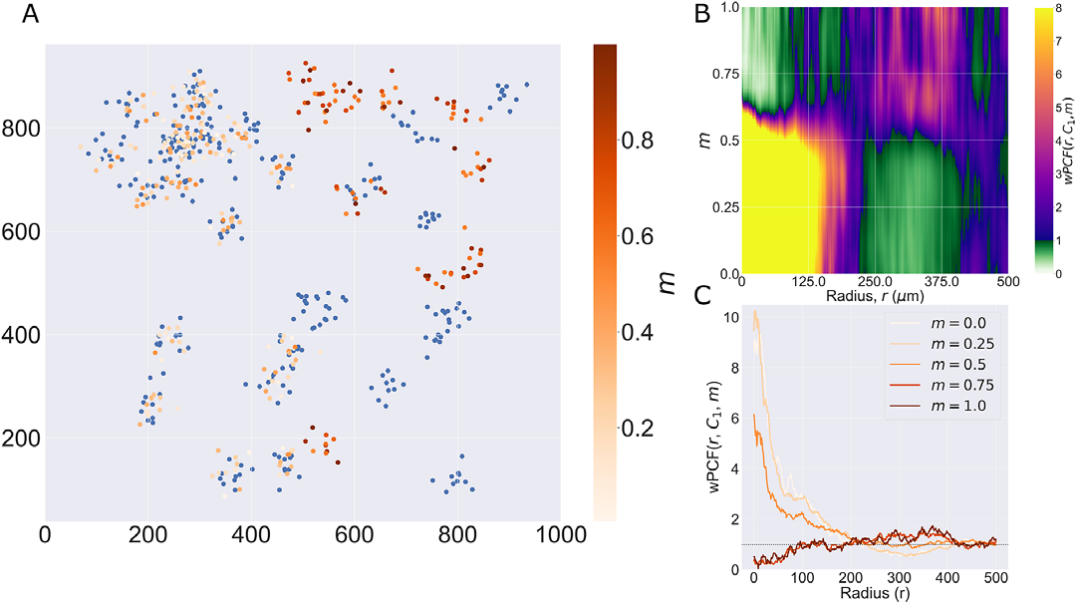
Motivating example III: weighted-PCF. (a) Synthetic dataset I: the same point pattern from Figure 2, now shown with the continuous mark 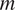 associated with cells of type 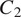 . Recall cells of type 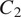 with 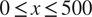 have 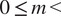 0.5, while those with 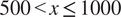 have 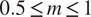 . (b) The wPCF, 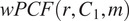 , for the point pattern in panel a identifies differences in clustering between cells of type 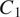 and cells of type 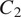 with marks above or below 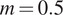 . (c) Cross sections of the wPCF in panel b. These plots distinguish the strong clustering of cells of type 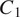 with cells of type 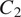 that have 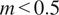 and their weak exclusion from cells of type 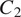 that have 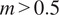 .

Figure 4b shows 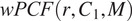 for the point pattern in Figure 4a, with cross sections of the wPCF shown in Figure 4c. For a given target value 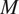 , the cross sections of the wPCF can be interpreted in the same manner as the cross-PCF or PCF. Figure 4b identifies two types of correlation in the data, each associated with different values of 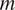 . For 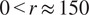 , there is strong short-range clustering between cells of type 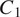 and cells of type 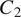 with 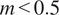 , with weak short-range exclusion up to this length scale for 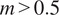 . Since cells on left-hand side of the domain have 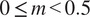 , and those on the right-hand side have 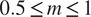 , this effect is consistent with the information from the cross-PCF and TCM above. One advantage of visualizing the wPCF as a heatmap (Figure 4b) is that it identifies threshold values of 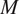 at which the nature of the cell–cell correlations changes, as demonstrated in Reference (48).

## 3. Results

**Figure 5.**
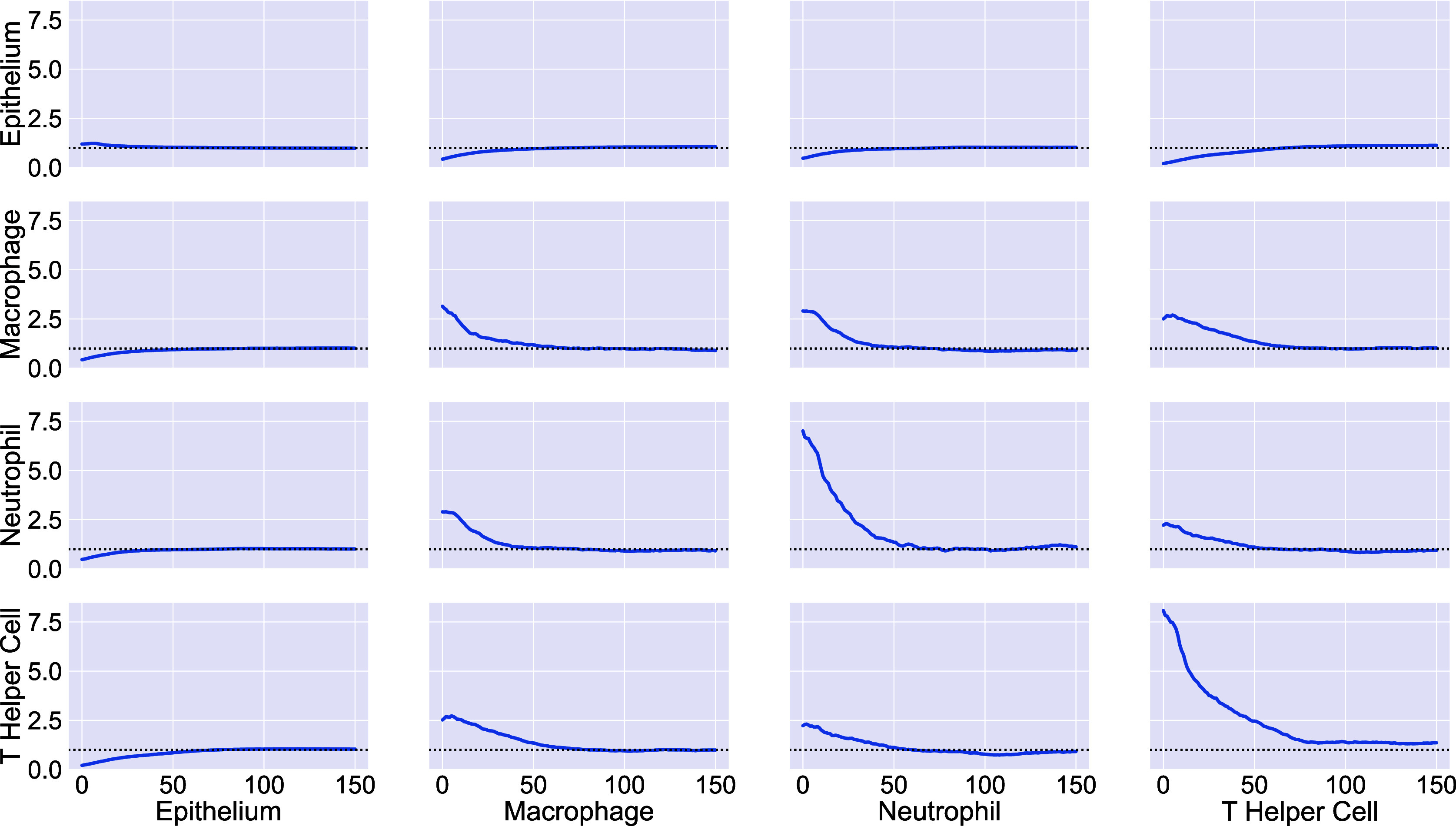
Cross-PCFs for pairwise combinations of cell types in the ROI. Cross-PCFs for pairs of cell types from the ROI. We observe exclusion between epithelium and all immune cell subtypes, and strong pairwise correlation with macrophages, neutrophils, and T helper cells on short length scales. Results involving regulatory and cytotoxic T Cells are omitted as their cell counts are low in this ROI.

In this section, we illustrate the utility of the TCM, NCF, and wPCF through their application to an ROI from a multiplex IHC image of a murine colorectal carcinoma (see the Methods section for details, and Section S1 of the Supplementary Material for similar analyses of three additional ROIs). Figure 5 shows the cross-PCFs that describe the pairwise correlations between all cell types present in the data. Due to the low numbers of cytotoxic and regulatory T cells, we focus subsequent analyses on relationships between epithelium and T helper cells (two abundant cell types that are spatially anticorrelated) and on T helper cells and macrophages (the most abundant immune cell subtypes, which are spatially correlated). When applying the NCF, we include neutrophils as a third immune cell subtype which colocalizes with T helper cells and macrophages.

### 3.1. Cross-PCFs and TCMs identify colocalization and exclusion in cell center data

**Figure 6.**
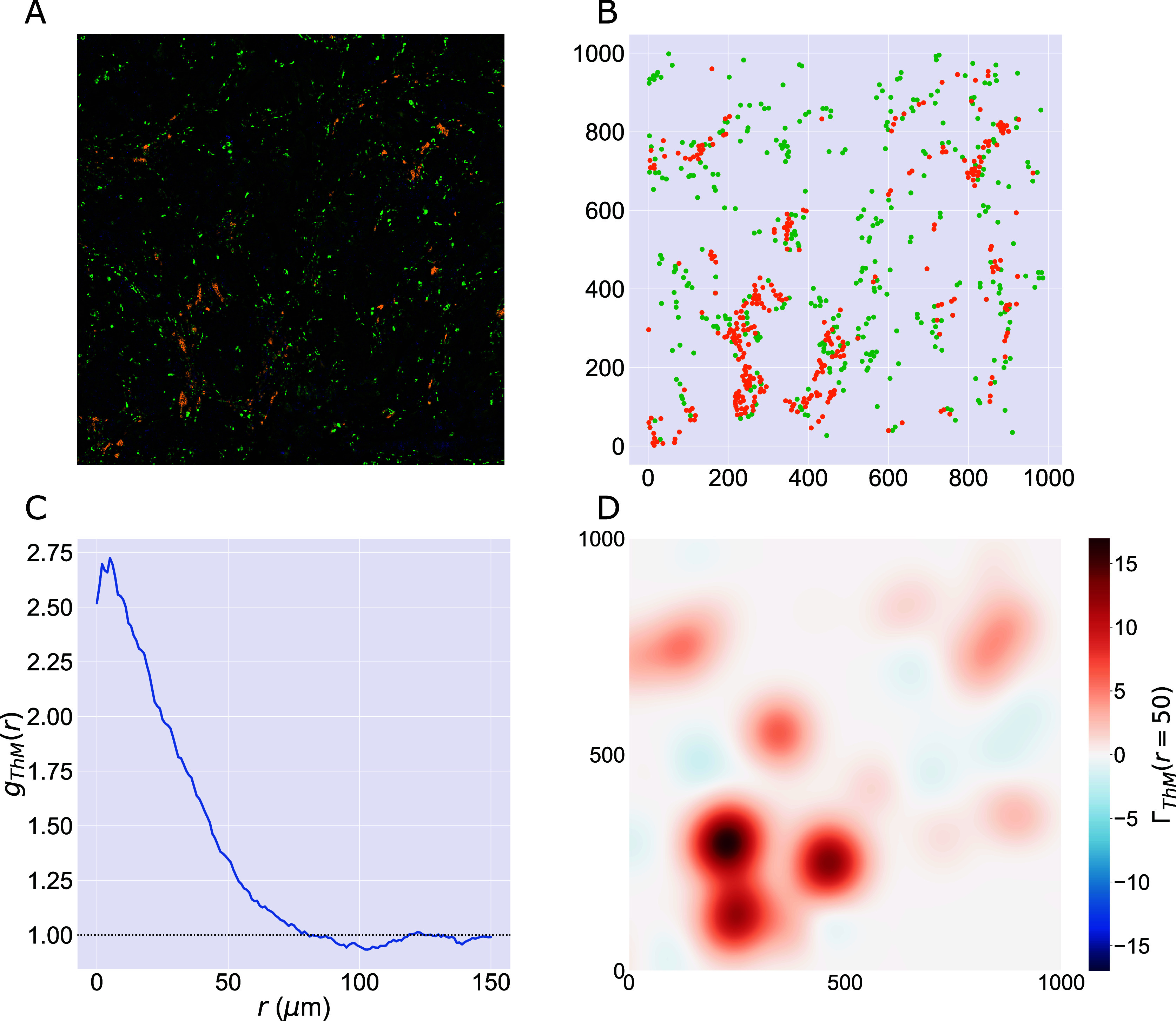
PCF and TCM for positively correlated cell types. (a) Locations of T helper cells (CD4+, orange) and macrophages (CD68+, green) in the ROI (with DAPI, blue). These cell types colocalize in the tissue between epithelial cell islands. (b) Cell centers identified as T helper cells (orange) and macrophages (green). (c) Cross-PCF for T helper cells to macrophages, 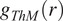 . These cell types are spatially colocated over a wide range of distances, that is, 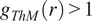 for 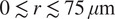 . (d) TCM for T helper cells to macrophages, 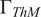 , for 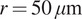 . Red regions indicate colocalization of the cell types in stromal regions, while blue regions correspond to isolated T helper cells.

We first consider T helper cells (Th) and macrophages (M), which are shown to be colocalized from the cross-PCFs in Figure 5. Figure 6a shows the channels of the multiplex image that correspond approximately with T helper cells (CD4+, orange) and macrophages (CD68+, green); the cell centers of these cell populations within the ROI are shown in Figure 6b (see Figure 1 for other cell locations). In this ROI, both T helper cells and macrophages are predominantly found in the stromal tissue between islands of (cancerous) epithelial cells, leading to positive spatial correlation on short length scales ( 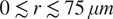 ).

Colocalization is clearly identified by the cross-PCF in Figure 6c: for 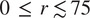 , 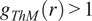 , indicating clustering between the cells of up to 2.75 times greater than expected under CSR, on length scales up to approximately 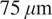 (a distance approximating the width of the stromal region that separates epithelial clusters).

Figure 6d shows 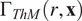 for 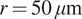 . This permits the clustering identified by the cross-PCF to be mapped onto the ROI, revealing subregions in which T helper cells are spatially colocated with, or excluded from, macrophages. We observe strong clustering in stromal regions, with islands of weak exclusion where isolated T helper cells are present. We conclude that, while T helper cells typically colocalize with macrophages, certain subregions of the ROI that contain T helper cells have low numbers of macrophages within a 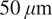 radius. Further, these subregions do not contribute significantly to the overall correlation of T helper cells with macrophages in the cross-PCF.

**Figure 7.**
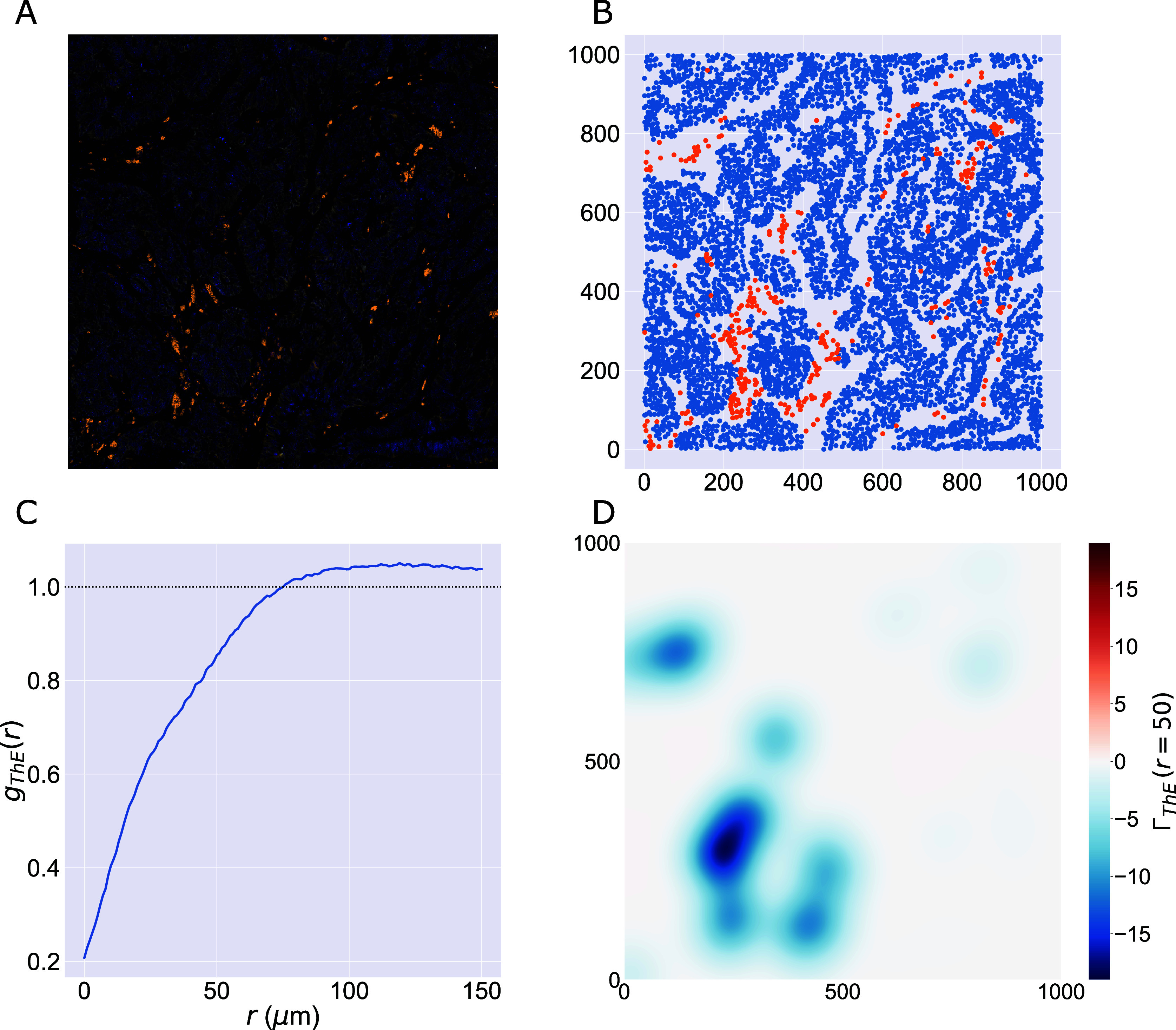
PCF and TCM for negatively correlated cell types. (a) Locations of T helper (CD4+, orange) and epithelial cells (E-cadherin+, white) in the ROI (with DAPI, blue). Epithelial cells exist in clumped “nests,” with T helper cells restricted to the stromal regions between them. (b) Cell centers of T helper cells (orange) and epithelial cells (blue). (c) PCF for T helper cells to epithelial cells, 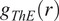 . We observe strong spatial exclusion, as 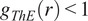 for 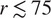 . (d) TCM for T helper cells to epithelial cells, 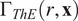 , for 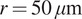 . The blue regions showing strong exclusion indicate subregions of the ROI which are devoid of epithelial cells. The strongest signals occur where T helper cells are organized in large clusters, while regions with few T helper cells do not contribute significantly to the cross-PCF.

In Figure 7, we focus on T helper (Th) and epithelial cells (E), which are shown to be anticorrelated by the cross-PCFs in Figure 5. In Figure 7c, the cross-PCF 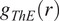 shows exclusion for 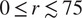 , with the strongest exclusion occurring on small length scales. This exclusion is also identified by 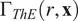 in Figure 7d, which shows that the cross-PCF is dominated by strong exclusion from T helper cells in the stromal islands between epithelial cells as expected. The contributions from T helper cells outside the stromal regions (e.g., those in the lower right quadrant of the ROI) are negligible compared to those in the lower left quadrant, due to the large number of T helper cells in that subregion.

### 3.2. NCFs identify spatial correlations between three cell types simultaneously

**Figure 8.**
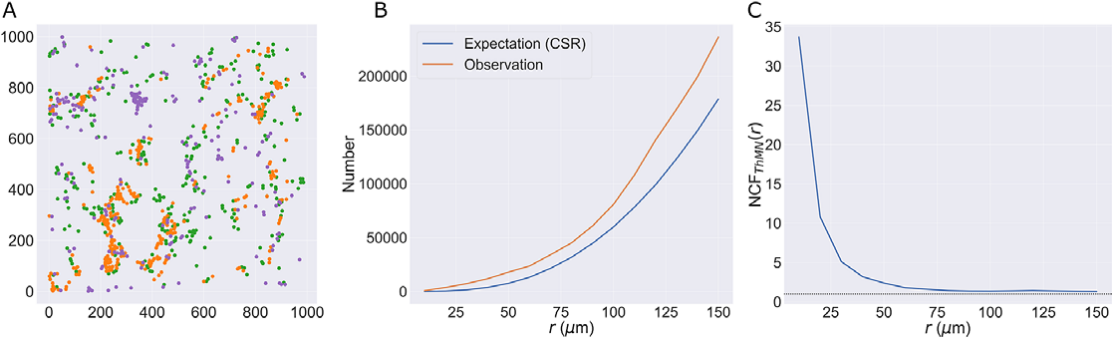
The NCF identifies spatial colocalization between three cell types. (a) Locations of T helper cells (orange), macrophages (green), and neutrophils (purple) extracted from the ROI. All three cell types are found in stromal regions, while macrophages and neutrophils are more likely to be observed within the epithelial islands (e.g., in the top left corner). (b) Expected and observed numbers of circles of radius 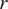 . (c) NCF obtained by computing the ratio of the curves in panel b, 
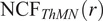
 . For 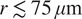 , neutrophils, macrophages, and T helper cells are colocalized within a circle of radius 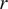 more often than would be expected under CSR.

Figure 8 shows the NCF for macrophages, T helper cells, and neutrophils (spatial locations shown in Figure 8a). We calculate the smallest circles enclosing each triplet containing one of each cell type, and note their radii. Figure 8b compares the number of circles with radius 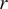 observed in the data, with the expected number if macrophages, neutrophils and T helper cells are randomly distributed (obtained via simulation as described in the methods, for 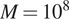 ). More circles are observed than expected under CSR. By taking the ratio of the curves in Figure 8b, we generate the NCF in Figure 8c. The NCF shows that triplets comprising a macrophage, a neutrophil and a T helper cell are up to 35 times more likely to cluster within a neighbourhood of radius 0– 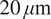 than would be expected if the cells were randomly distributed. We conclude that these cell types are frequently found together.

### 3.3. The wPCF identifies correlations without classification or segmentation

Recall that in order to apply the PCF, cross-PCF, TCM, and NCF, the multiplex imaging data must be segmented and then classified to identify cell centers and assign them categorical labels (or cell types). We now show how the wPCF can be applied directly to multiplex imaging data to identify spatial correlations, without segmentation or classification.

**Figure 9.**
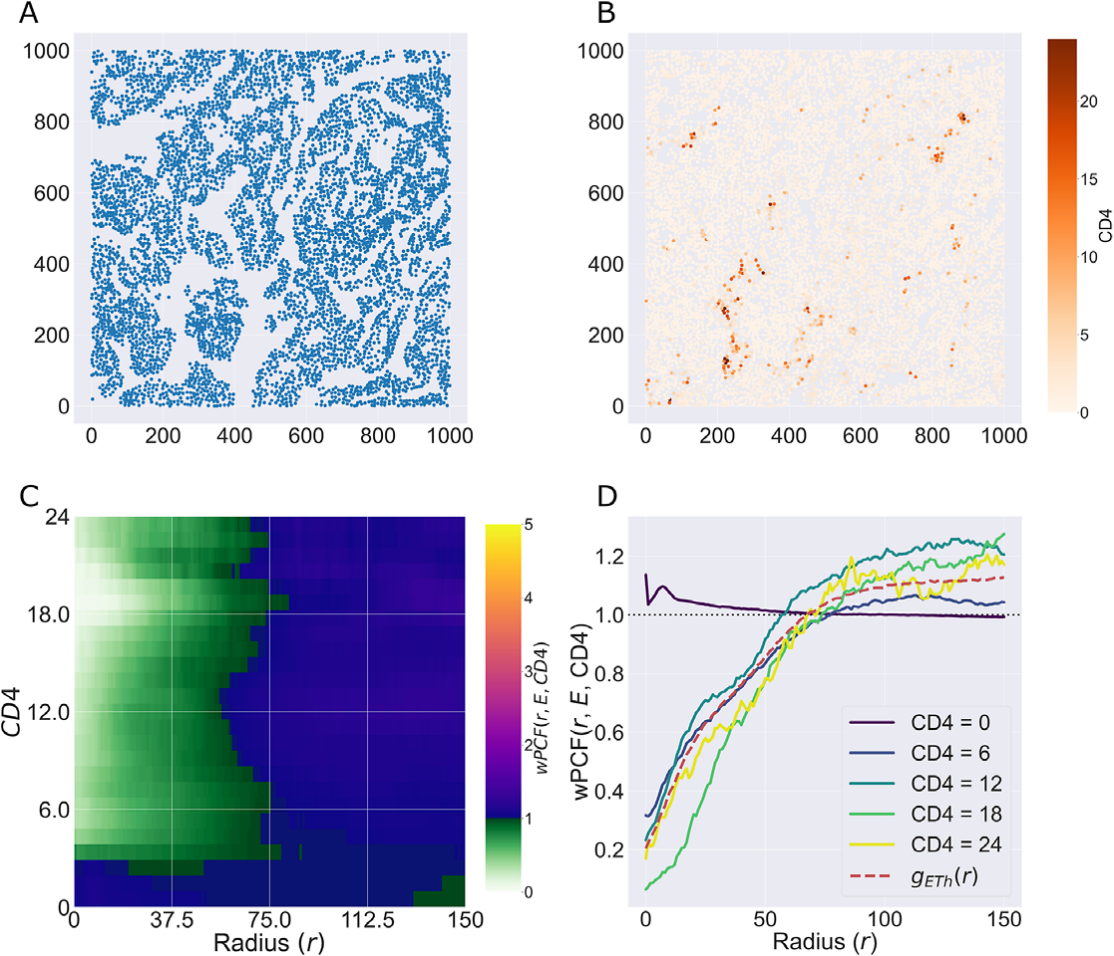
The wPCF identifies correlation between epithelial cells and cells with different CD4 expression levels. (a) Epithelial cell centers. (b) Cell centers labeled according to the average CD4 stain intensity within each cell. (c) wPCF ( 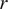 , 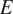 , CD4), showing clear qualitative and quantitative differences in colocalization with epithelial cells as CD4 expression levels vary. (d) Cross sections of the wPCF in panel c. Points with low CD4 expression have a different pattern of correlation than those with higher expression. The profile for cells with high CD4 expression corresponds to the cross-PCF 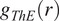 , calculated for cells which have been manually classified as T helper cells (red dashed line). Cells with low CD4 intensity colocalize with epithelial cells, likely due to many epithelial cells having low CD4 expression. Cells with higher expression of CD4 are anticorrelated with epithelial cells for 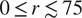 .

Figure 9 demonstrates that the wPCF can identify correlations when some cells are not classified. Rather than specifying a threshold value of the CD4 marker intensity to identify CD4+ cells, we instead view the average CD4 intensity of each cell as a continuous mark. Figure 9a shows epithelial cells determined by specifying a threshold, while Figure 9b shows all cells labeled according to their average CD4 intensity. The wPCF calculated in panel c shows that the spatial positions of cells with low CD4 intensity differ from those with high CD4 expression (with 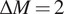 in Equation (11)). Figure 9c,d shows that cells with mean CD4 intensity below approximately 4 are not strongly correlated with epithelial cells. However, for larger values of CD4 intensity, the profiles of the wPCF are in good agreement with the cross-PCF 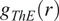 (shown as a red dashed line in Figure 9d).

**Figure 10.**
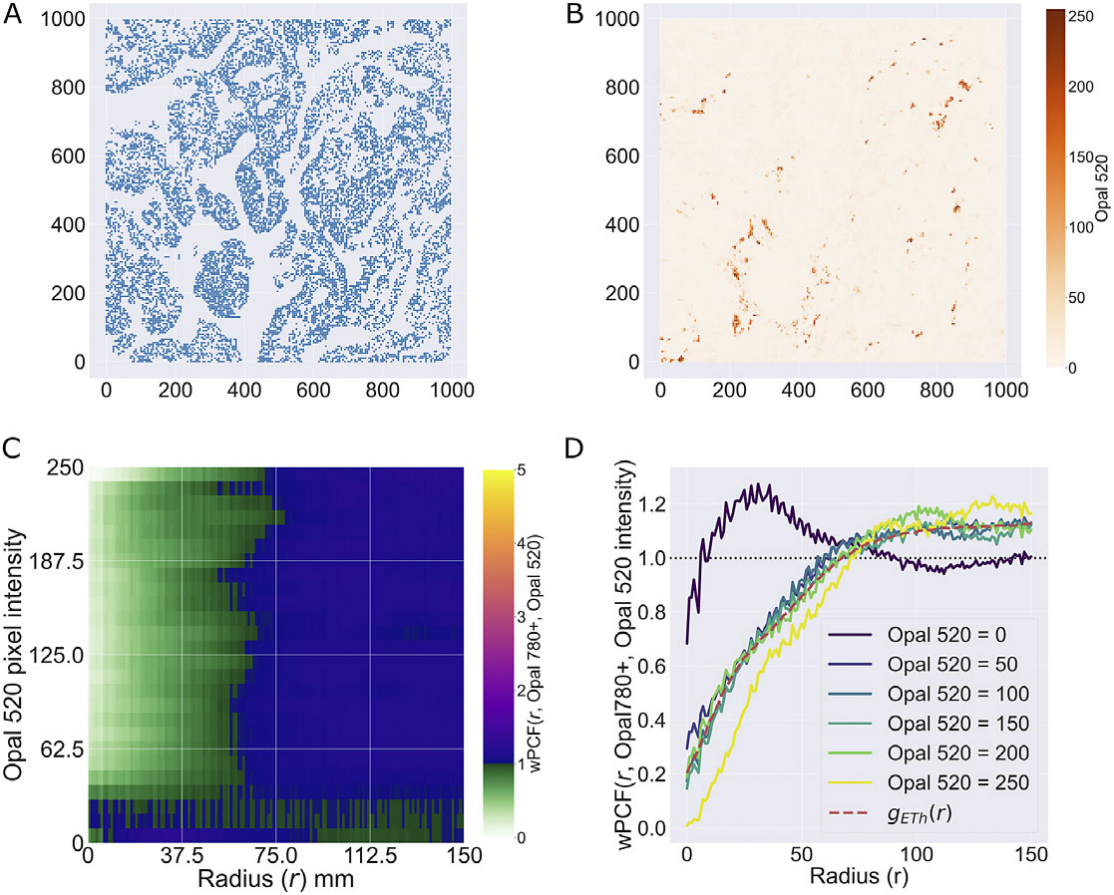
wPCF identifies correlation between epithelial cells and pixels with varying CD4 expression. The results from Figure 9 are recovered when the wPCF is calculated from points sampled from the original multiplex image using a regular 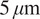 lattice, showing that the spatial correlation between T helper cells and epithelial cells can be identified without segmentation or classification. (a) Pixel intensities of the Opal 520 marker (associated with CD4), sampled across the ROI on a regular 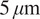 lattice. (b) Pixels marked as Opal 780 positive (associated with epithelial cells), determined via thresholding, sampled across the ROI on a regular 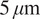 lattice. (c) wPCF describing correlation between pixels positive for Opal 780 and the pixel intensity of Opal 520. (d) Cross sections of the wPCF in panel c have the same shape as the cross-PCF in panel d for pixels with high CD4 intensity.

Finally, in Figure 10, we show that application of the wPCF to multiplex images, without cell segmentation or classification, can identify spatial correlation. Panels a and b show points from a regular lattice sampled from the multiplex image of the ROI, at a resolution of 1 point every 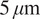 . In panel a, points are labeled according to a thresholded value of the epithelial cell marker, while in panel b, they are labeled according to the CD4 intensity at that pixel (note we use the notation “Opal 520,” the marker associated with CD4 cells, to distinguish these raw pixel intensities from the mean pixel intensities used in Figure 9). The wPCF which compares these marks is shown in panel c, and is in good qualitative and quantitative agreement with the wPCF from Figure 9 (with 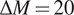 in Equation (11)). We conclude that applying the wPCF directly to pixels and stain intensities can identify the same spatial patterns of clustering and exclusion as those identified by the cross-PCF, without cell segmentation or classification.

## 4. Discussion

Multiplex images contain a wealth of spatial information, and have the potential to greatly increase the information that can be extracted from histological samples. Each image provides a high-resolution map of cell locations across tissue samples that may contain millions of cells, together with detailed information about their phenotypes and morphology. As multiplex images become more widespread and as digital tools for their visualization and analysis improve, the demand for automated methods that can extract detailed spatial information from them is increasing. Such methods should be agnostic to the technology used to generate the images, the disease under investigation, and the particular markers with which the sample has been stained.

Many existing methods can extract information from multiplex images. One popular approach involves using AI or machine learning approaches to identify correlations between features extracted from multiplex images and clinically relevant features such as disease progression. AI methods can be extremely powerful, but are not ideally suited to all situations. In particular, an AI algorithm may require vast numbers of images for use as training data. Further, the tissue type and panel of markers chosen for staining should be consistent across the training data, thereby reducing the applicability of the algorithm to samples from different diseases (e.g., an algorithm trained on multiplex images of immune cells in colorectal cancer cannot reliably be applied to images of immune cells in prostate cancer, or to images of stromal cells in colorectal cancer). AI methods can sometimes lack interpretability, making it difficult to understand which features of an image an algorithm is using and to understand when errors are likely to arise.

On the other hand, a range of statistical and mathematical methods can also describe features of multiplex images in an interpretable way. These methods may derive from a range of disciplines, such as network science, TDA, and spatial statistics. They provide quantitative descriptions of specific spatial features of an image; for example, ecological analyses may describe correlations in cell counts across subregions of an ROI with a fixed area, quantifying the strength of local correlations.^(^^29^ ^)^ Existing metrics have typically been developed to address a specific problem. As a result, multiple methods may be used to describe the same features of a point pattern. For instance, the field of spatial statistics encompasses a range of methods designed to identify correlations in point patterns, with specialized tools to address specific use cases. The PCF has been specialized to account for interactions between multiple classes of point (the cross-PCF), points generated from processes that vary across a region (inhomogeneous-PCF^(^^35^ ^)^), or points labeled with continuous marks (mark correlation functions, weighted-PCFs). Such metrics can provide detailed information about the spatial structure of multiplex images, even though they may have been developed for other types of data. In order to understand multiplex images using quantitative metrics, we propose the application of multiple statistics (which may derive from different mathematical fields), designed to quantify specific properties of the image.

In this paper, we have focused on three methods for extending the PCF that have been specifically designed for application to multiplex medical images. Each is applied here for the first time to multiplex IHC images from the Vectra Polaris system, in order to illustrate how they address limitations in the PCF. We now summarize each method in turn, focusing on their strengths and weaknesses.

### 4.1. Topographical correlation map

The TCM can visualize spatial correlations between pairs of cell populations across an ROI, highlighting subregions of strong positive or negative correlation that can be difficult to identify by visual inspection. The TCM can be calculated without requiring the user to estimate the intensity of an inhomogeneous point process; rather, it identifies subregions in which local interactions between the point patterns differ from those that would be obtained under homogeneous CSR. While we have used the TCM for visualization, it generates quantitative information which can be used for subsequent analysis. For example, the number and size of the local minima and maxima could be used as summary statistics to compare and classify images. The TCM can also be analyzed via a sub/super level-set filtration.^(^^60^ ^)^ This method from TDA can quantify spatial heterogeneity in heatmaps.

We note that, by design, the TCM is asymmetric (i.e., 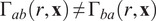 ). As such, care is needed when interpreting the TCM. In particular, while 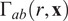 and 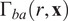 should coincide in regions of positive correlation, they may differ in regions of negative correlation. Further, regions where 
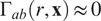
 cannot be used to infer the presence (or absence) of either cell type without consideration of other metrics (e.g., local cell densities).

### 4.2. Neighbourhood correlation function

The NCF identifies whether groups of three or more cells are found in a circular neighbourhood of radius 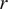 more or less frequently than expected under CSR. Since it requires distance calculations between 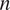 cell types, the computational complexity of the NCF is at least 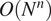 . This limits its potential application to WSIs or to identifying correlations between a large number of cell types simultaneously: although calculating each enclosing circle is fast,^(^^58^ ^)^ as the maximum number of circles which must be calculated is 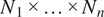 (where 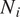 is the number of cells of type 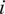 ) the computational effort involved increases rapidly as the total number of cells and number of cell types increase. As for the PCF, the runtime performance of the algorithm can be improved by calculating the NCF up to a maximal neighbourhood size of interest 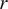 ; this reduces the number of 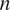 -wise maximum enclosing circles that must be calculated (any combination of points containing a pair separated by more than the length scale of interest can be immediately discarded). The NCF also relies on repeated sampling of random data to identify the expected number of neighbourhoods that would be observed under CSR. For a given region, this probability can be calculated in advance to an arbitrary level of precision, and becomes more accurate with more samples. However, more research is needed to determine the minimum number of samples needed to achieve a given accuracy.

The process of calculating the NCF suggests that in future work it could be adapted to produce a spatially-resolved map, similar to the TCM, which would indicate areas of an ROI in which 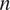 cells of interest colocalize. For example, Figure 3c,g suggest that placing a kernel on each neighbourhood which is weighted inversely to the radius of the circle would generate a landscape in which colocalization of multiple populations would be identified at local maxima.

### 4.3. Weighted-PCF

The wPCF generalizes the cross-PCF to data with continuous labels (e.g., cell centers that have not been classified into discrete categories, or to pixels which have not been segmented to find cell centers). As such it can be calculated without classification or cell segmentation preprocessing steps. However, this also increases the number of “parameters” required. In particular, the choice of weighting function determines the ratio of signal/noise identified by the wPCF and must be considered in advance (see Reference (48) for a detailed examination of the impact of varying the weighting function on the wPCF). The tuning parameters used to construct the wPCF are in some ways similar to those used to perform cell classification (e.g. threshold values for stain intensities). Since it requires careful choice of the weighting function and associated parameters, users should carefully consider the most appropriate choice of method for comparing their data, since depending on the context methods designed to directly compare random fields of intensities may be more appropriate than the point process setting (see, e.g., Reference (61) for a detailed comparison of some relevant pixel-based and object-based methods for quantifying colocalization).

There is considerable scope for developing approaches to interpret the wPCF. The heatmap that it generates can be analyzed using techniques similar to those discussed for the TCM above. Further, since the wPCF generates outputs comparable to a series of cross-PCFs with different target marks, it may be possible to define an analogue of the TCM in order to localize regions in which the populations of interest colocalize. For example, a similar kernel method to that defined for the TCM could be used, in which kernels are scaled both by strength of colocalization (as in the current implementation) and by the weighting of the point relative to the target mark in the wPCF.

It is also possible to use the outputs from the wPCF to create a vectorized “spatial signature” which can be used to cluster regions which have similar spatial structures.^(^^48^ ^)^ Such an approach could be used to automatically identify regions with similar spatial cellular interactions, or which contain spatial patterns associated with, for example, cancer progression or disease severity. Indeed, by vectorizing the spatial descriptors described within this paper the approach described in Reference (48) to identify such “spatial biomarkers” could be extended.

## 4.4. Conclusions

Multiplex images contain vast amounts of spatial information which can be exploited using quantitative techniques. The spatial statistics considered in this paper represent one approach to analyzing these data, and benchmarking studies that compare the efficiency and insight of different methods are needed. There are several challenges associated with applying methods based on spatial statistical analysis of point patterns, such as those described in this paper, to large regions, such as whole slide images, or images with large numbers of different cell types (e.g., 60+). One major limitation relates to their scaling as the number of cells increases (see Supplementary Material), since WSIs may contain millions of individual cells. Improvements in the efficiency of code implementation are likely to be needed in these cases, such as parallelizing calculations or restricting the number of pairwise distance calculations that must be performed by introducing distance thresholds.

The methods described in this paper were designed to exploit the spatial information contained in multiplex images. We note, however, that they can be applied to multiple imaging modalities and multiple diseases. Equally, each method can be applied to generic point cloud data from contexts outside of biology.

We have previously shown that combining spatial statistics can generate more comprehensive descriptions of point data than individual metrics alone.^(^^38^ ^,^^48^ ^)^ In future work, we will determine how complementary methods from mathematical fields such as spatial statistics, network science, and topology, can build upon this to provide a rigorous quantitative description of how data are spatially distributed.

## Supporting information

Bull et al. supplementary material Bull et al. supplementary material

## Data Availability

Python code for reproducing the results described in this paper, along with the relevant data, is available on github at https://github.com/JABull1066/ExtendedCorrelationFunctions.

## Supplementary material

The supplementary material for this article can be found at http://doi.org/10.1017/S2633903X24000011.
