# Supplementary material for: Extended correlation functions for spatial analysis of multiplex imaging data
Source: Biol Imaging. 2024 Feb 15;4:e2. doi: 10.1017/S2633903X24000011 (PMC10951806; doi:10.1017/S2633903X24000011)
Supplement: Bull et al. supplementary material [file S2633903X24000011sup001.pdf]

Supporting Information for: Extended correlation functions for spatial analysis of multiplex imaging data

Joshua A Bull, Eoghan J Mulholland, Simon J Leedham, Helen M Byrne

June 16, 2023

S1 Analysis of other regions of interest

In this section we present supplementary analyses (without additional commentary) for three additional 1mm $\times$ 1mm regions of interest from within the same KPN mouse presented in the main text. The same cross-PCFs, NCFs, TCMs and wPCFs as those found in the main text are presented below, and show the same general results presented in the main text.

Celltype	Marker	Region 1 - n	Region 2 - n	Region 3 - n
Epithelium	E-Cadherin	5122	5812	5737
Macrophage	CD68	549	287	467
T helper cell	CD4+ FoxP3-	965	91	666
Neutrophil	Ly6G	116	165	163
Cytotoxic T cell	CD8	163	14	130
Regulatory T cell	CD4+ FoxP3+	25	5	49

Table 1: Number of cells, n, for each supplementary region

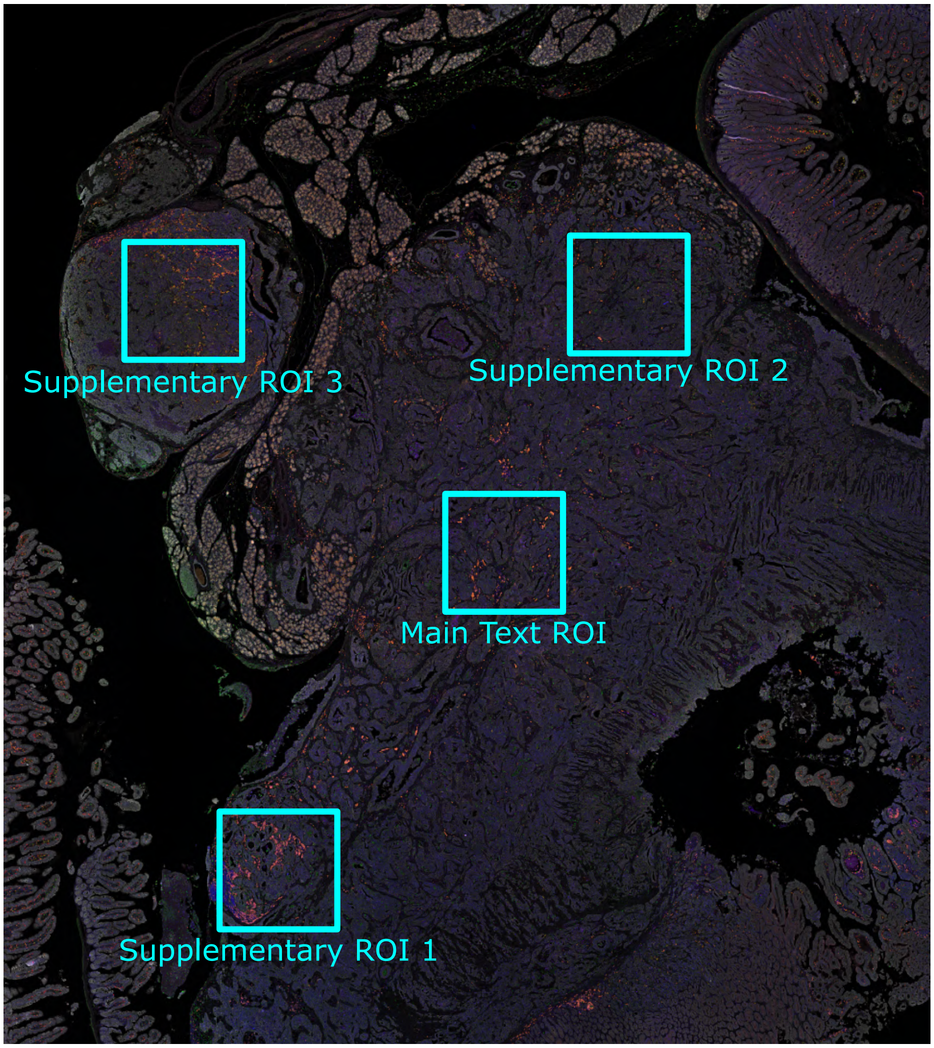

Figure S1: Location of 1mm × 1mm ROIs within the wider tissue context

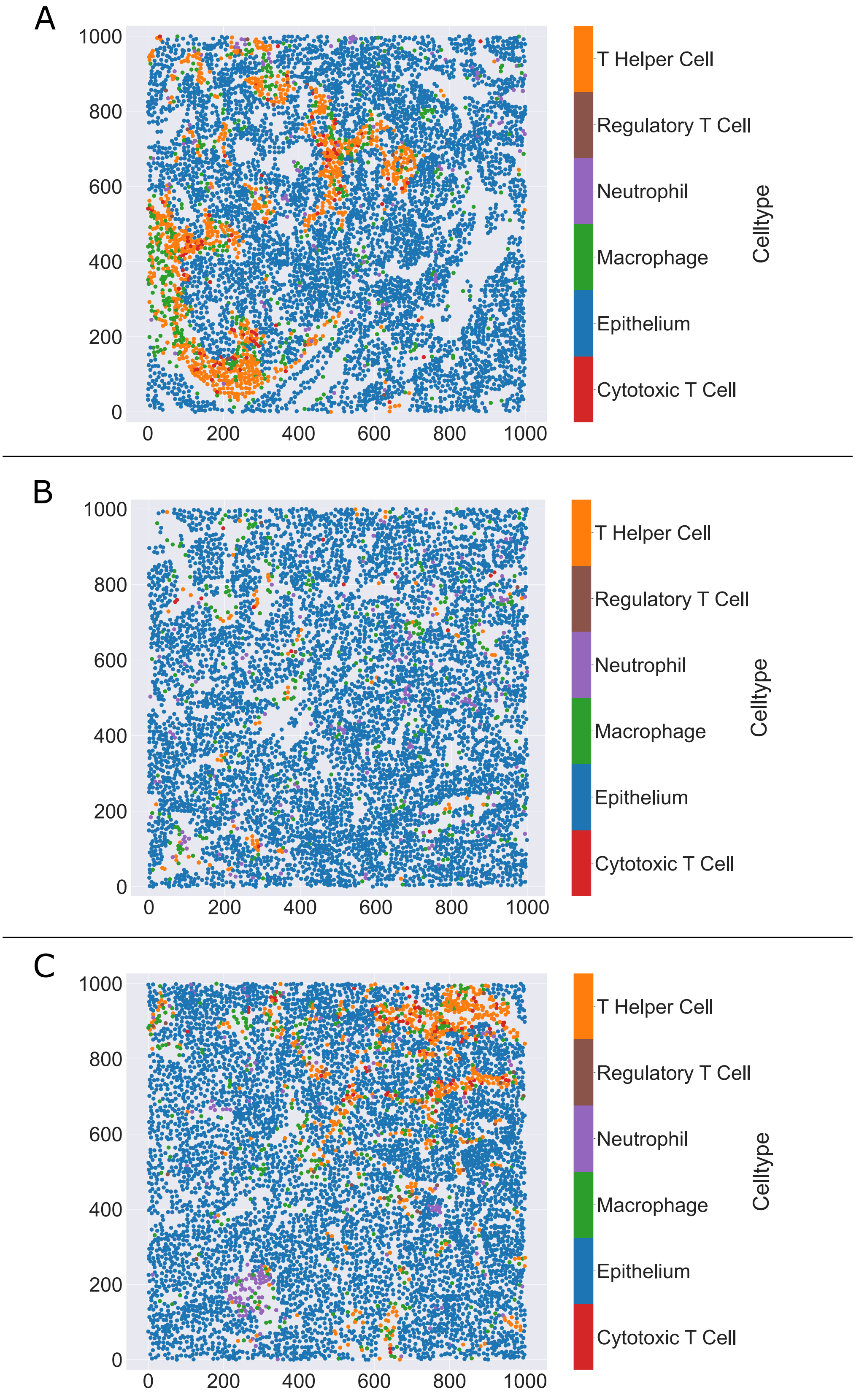

Figure S2: Cell centre locations for supplementary ROIs 1 (A), 2 (B) and 3 (C)

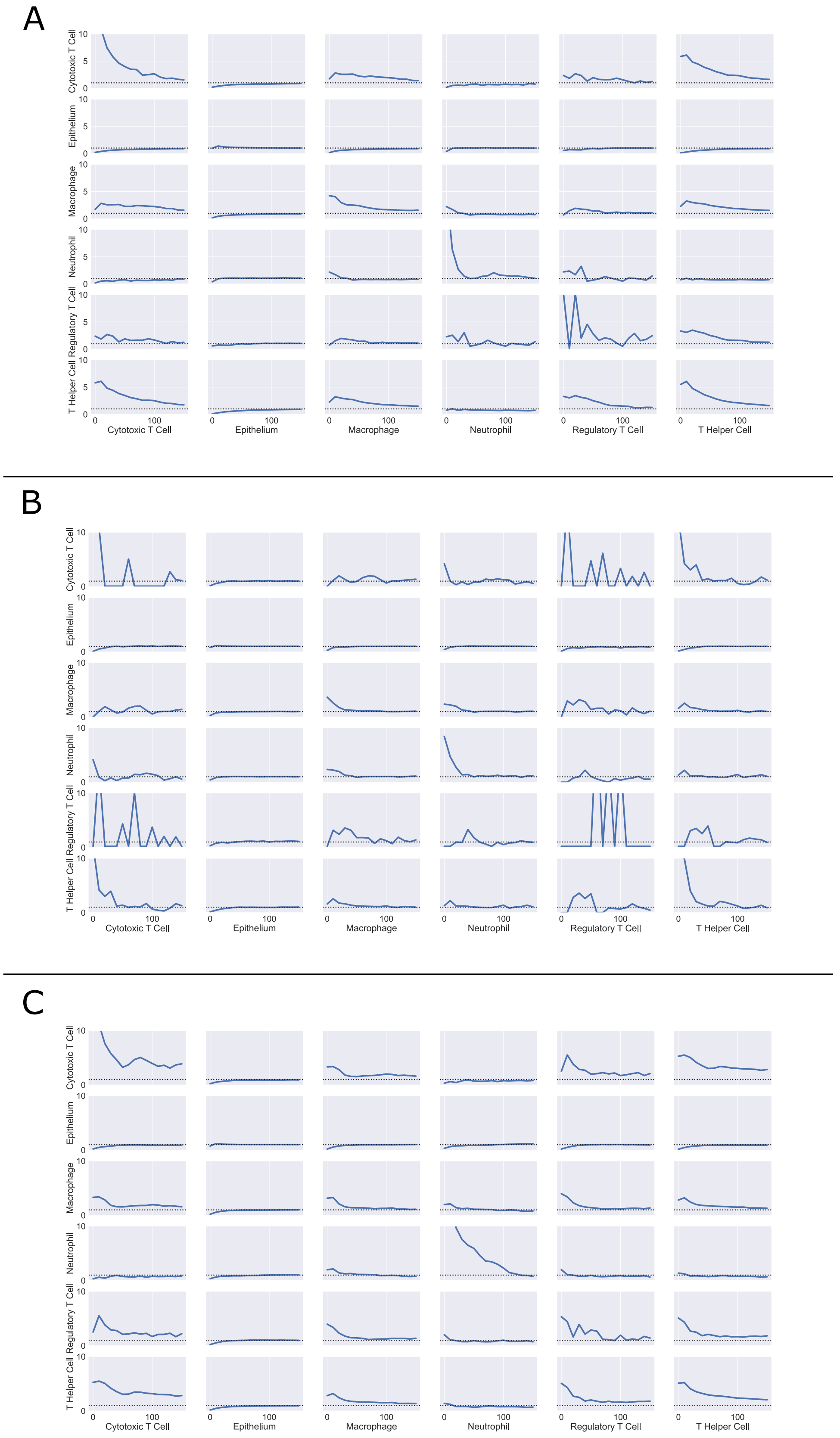

Figure S3: All cross-PCFs from supplementary ROIs 1 (A), 2 (B) and 3 (C)

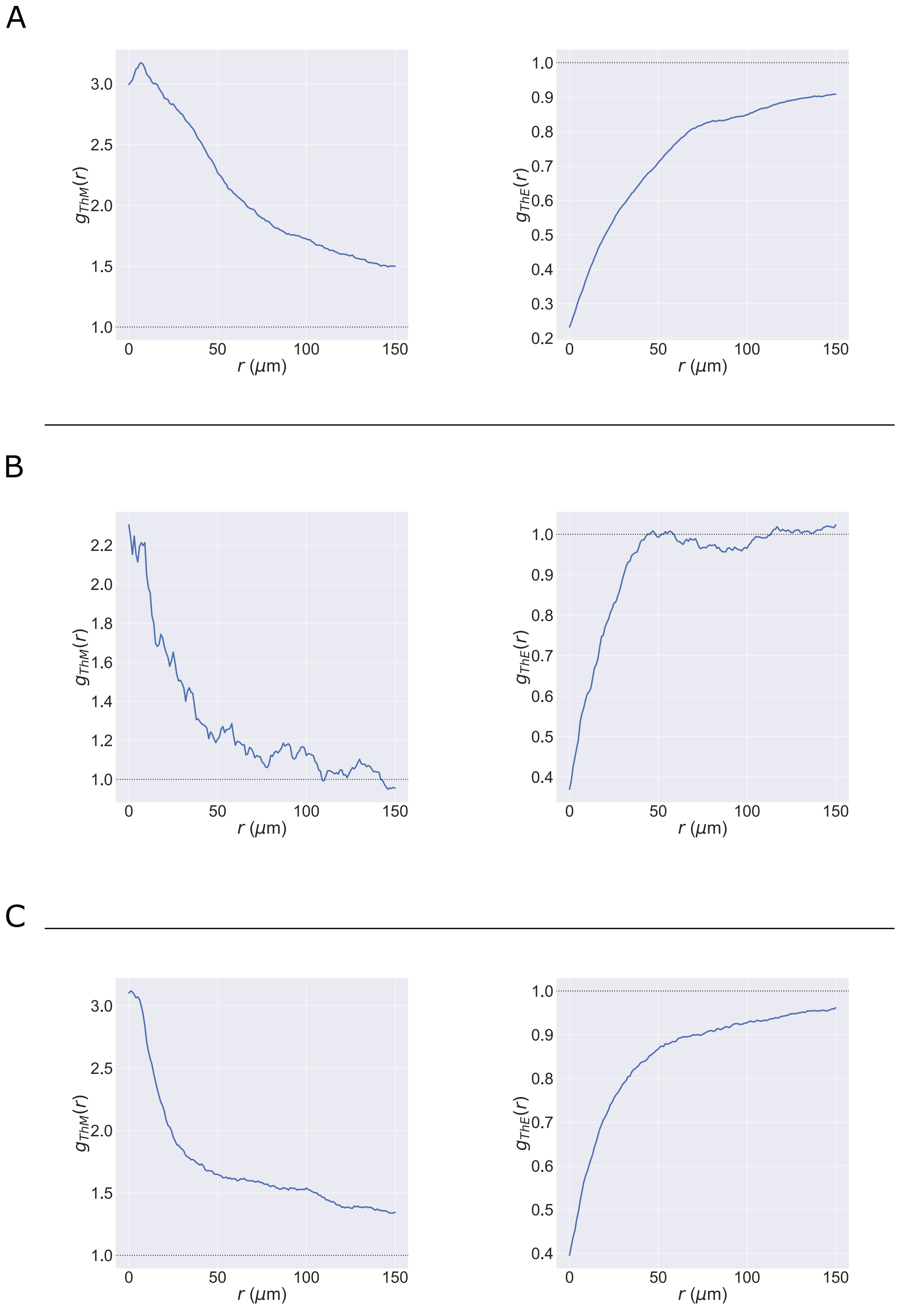

Figure S4: Cross-PCFs between T Helper Cells and Macrophages (left) / Epithelium (right) from supplementary ROIs 1 (A), 2 (B) and 3 (C)

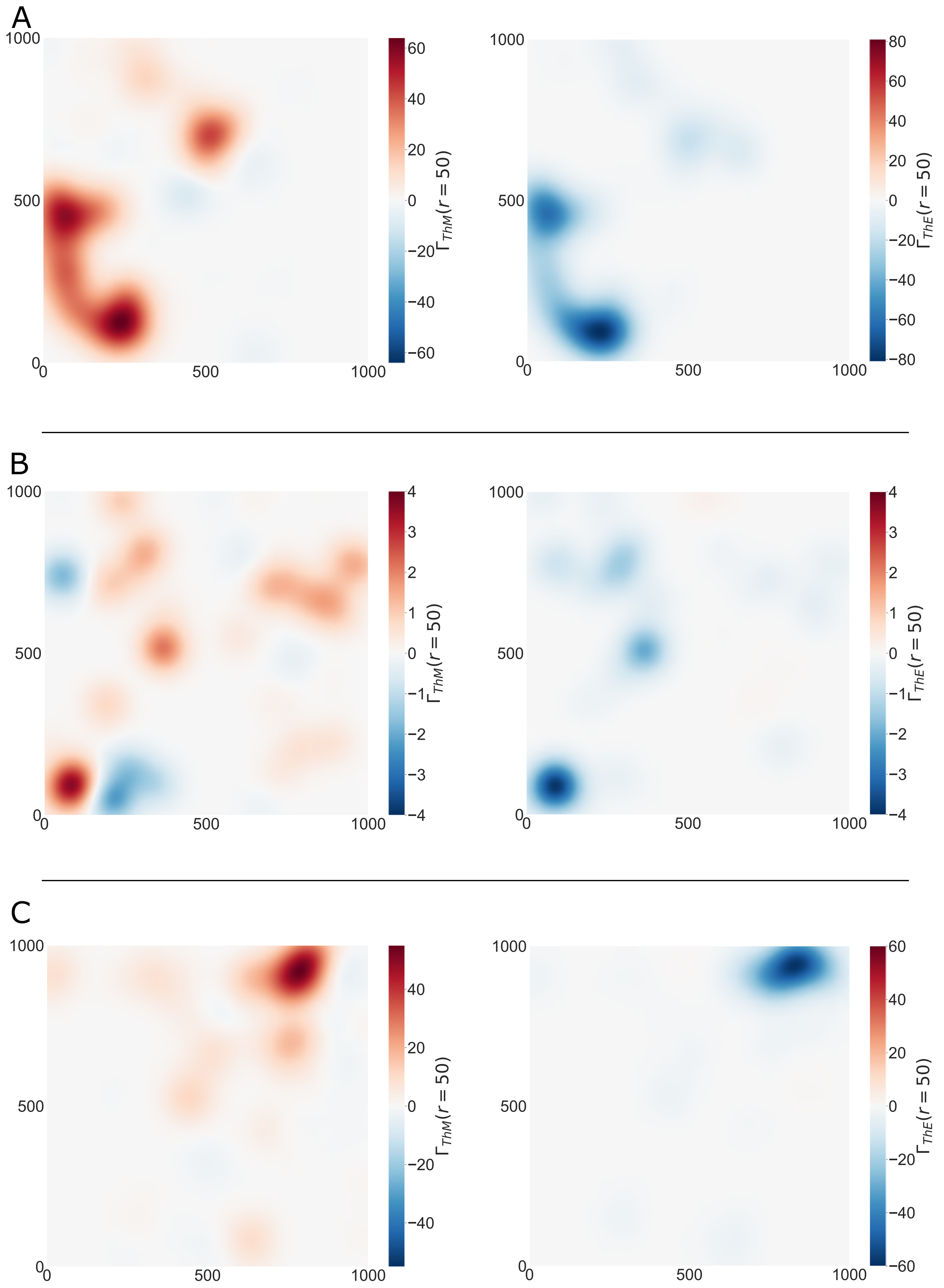

Figure S5: TCMs between T Helper Cells and Macrophages (left) / Epithelium (right) from supplementary ROIs 1 (A), 2 (B) and 3 (C)

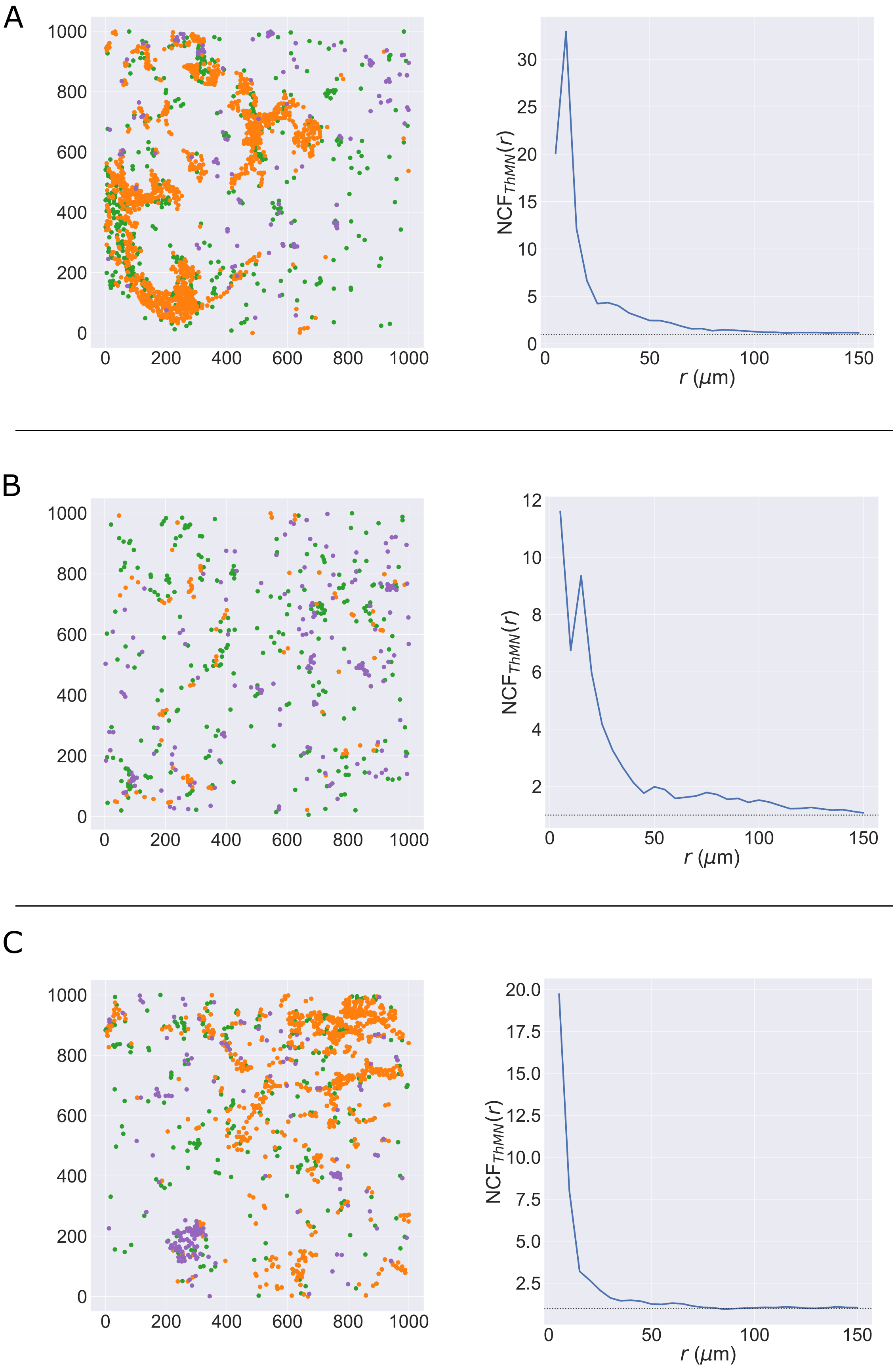

Figure S6: Left: locations of T Helper cells (orange), Macrophages (green) and Neutrophils (purple) from supplementary ROIs 1 (A), 2 (B) and 3 (C). Right: NCF between T Helper cells, Macrophages and Neutrophils from supplementary ROIs 1 (A), 2 (B) and 3 (C).

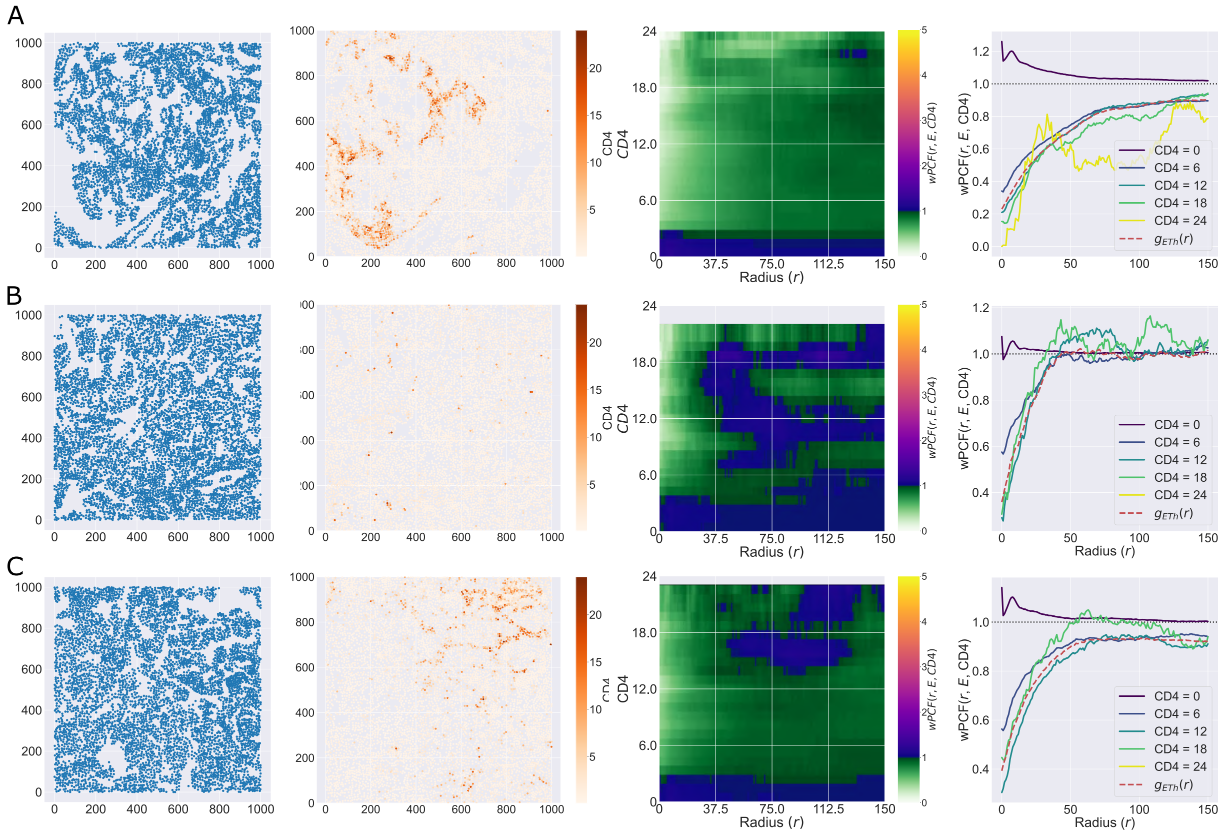

Figure S7: From left: i) locations of epithelial (cancer) cells; ii) all cells labelled according to CD4 intensity; iii) wPCF(r, E, CD4); iv) cross-sections of wPCF(r, E, CD4) at fixed values for the CD4 intensity. From supplementary ROIs 1 (A), 2 (B) and 3 (C).
